# Annexin A4 Deficient Ventricular Myocytes Display Advanced Hypertrophy and Electrical Remodeling With Chronic β‐Adrenoceptor Stimulation

**DOI:** 10.1096/fj.202600257R

**Published:** 2026-09-02

**Authors:** Florentina Pluteanu, Alexander Heinick, Manuel Domnik‐Lehnert, Frank U. Müller, Christina Hermes

**Affiliations:** ^1^ Department of Anatomy, Animal Physiology and Biophysics, Faculty of Biology University of Bucharest Bucharest Romania; ^2^ Institute of Pharmacology and Toxicology University Hospital Münster Münster Germany

**Keywords:** action potential, annexin A4, cardiomyocytes, ion channels, isoprenaline, ventricular hypertrophy

## Abstract

Annexin A4 (A4) is a negative modulator of adenylyl cyclase type 5 (AC5) with increased expression in failing human hearts. Here, we investigated whether A4 deficiency contributes to cardiac electrical and structural remodeling induced by chronic stimulation of β‐adrenergic receptors (βAR). A4‐deficient mice (gene trapped, GT) and wild‐type (WT) were infused for 7 days with isoprenaline (ISO) or NaCl as control. Myocytes of ISO‐treated GT (GT_ISO_) displayed more hypertrophy, increased action potential duration, reduced K^+^‐current *I*_to_, preserved L‐type Ca^2+^ current *I*_CaL_ with a negative shift of voltage dependence of activation, in line with increased AC/cAMP/PKA signaling, and increased NCX1 versus WT_ISO_. At the molecular level, mRNA levels for *Kcnd3*, *Kcnip2*, *Cacna1c* decreased at unchanged protein levels of Kv4.2, Kv4.3, KChIP2, α1C, independent of genotype, suggesting posttranslational modifications of the channels underlying *I*_to_ and *I*_CaL_. Chronic ISO‐induced βAR desensitization and redox stress were confirmed by decreased cAMP production and lower mRNA levels of *Adrb1*, *Adcy5/6*, and *Sod2*, and reduced response to acute ISO, with no additional genotype‐dependent effects on calcium handling or contractility. Nevertheless, GT_ISO_ cardiomyocytes retained a greater cAMP response to acute ISO, in line with AC5 disinhibition and preserved β2AR and Gαs/i, suggesting genotype‐dependent differences in β‐adrenergic signaling under stress. mRNA levels of *Anxa4* were increased in hypertrophied WT_ISO_ vs. normal WT_NaCl_ hearts, supporting a protective role for A4. Overall, the advanced remodeling in A4‐deficient myocytes detected in response to chronic βAR stimulation proposes using A4 peptide as a therapeutic tool to prevent the progression of cardiac electrical remodeling.

## Graphical abstract

Annexin A4 (A4) inhibits adenylyl cyclase 5 and may protect the heart during chronic β‐adrenergic stress. After 7 days of isoprenaline, A4‐deficient mice showed greater cardiomyocyte hypertrophy, prolonged action potentials, reduced Ito, preserved *I*_CaL_ with altered activation, increased NCX1, and enhanced acute cAMP responsiveness compared with wild type. Chronic isoprenaline induced β‐adrenergic desensitization in both genotypes. Increased Anxa4 expression in wild‐type hearts supports a protective role against electrical remodeling, suggesting A4 as a potential therapeutic target in cardiac disease.

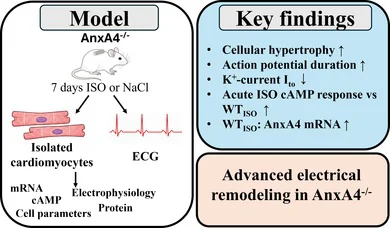

## 1 Introduction

Cardiac structural and electrical remodeling in response to pathologic beta‐adrenergic receptor (βAR) stimulation involves largely intracellular mechanisms sensitive to fluctuations of Ca^2+^ levels. Disturbances of Ca^2+^ homeostasis regulation in cardiomyocytes have been directly linked to the onset of arrhythmias and the progression of heart failure [1]. Intracellular Ca^2+^ increases as a result of influx from the extracellular space via voltage‐dependent Ca^2+^ channels and release from the sarcoplasmic reticulum via the ryanodine receptor type 2 (RyR2). The amount of free diastolic Ca^2+^ relies on the exclusion mechanisms via sarcoplasmic/endoplasmic reticulum Ca^2+^ ATP‐ase (SERCA2a) and Na^+^/Ca^2+^ exchanger (NCX1), as well as on the Ca^2+^‐buffering capacity of the cytosol. Annexins are such proteins capable of buffering intracellular Ca^2+^. Annexins bind Ca^2+^ in order to attach tighter to plasmalemmal phospholipids, where they mediate and modulate membrane delimited physiological activities such as exocytosis [2, 3, 4, 5] membrane repair [6, 7] or ion channel and receptor regulation [8, 9]. In cardiomyocytes, both Ca^2+^‐buffering function and the interaction with proteins of the plasmalemma such as NCX1 [10, 11] or endomembrane proteins like the RyR [10, 12] support the putative involvement of annexins in Ca^2+^ homeostasis during cardiac pathology.

The annexin family consists of 12 proteins (A1–A13, where A12 gene is unassigned) [13, 14, 15] that share a central hydrophobic core and differ at the level of N‐terminal regions. The calcium binding sites are localized to the core region which interacts with its own N‐terminus segment, where specific sites for phosphorylation and protein interaction are located [15, 16, 17]. Annexins bind Ca^2+^ with *K*_d_ values between 4 (A6) and 1000 (A5) μM in the absence of phospholipids, and *K*_d_ decreases significantly in the presence of phospholipids. The number of Ca^2+^‐binding sites is between two and four per annexin molecule [18]. The *K*_d_ calculated for A4 in the absence of phospholipids was 25 μM [18, 19] while isothermal titration calorimetry experiments identified 4 Ca^2+^ binding sites in the human A4 structure [20], placing A4 among intracellular Ca^2+^ buffers. Annexins A2, A5, and A6 were considered the most abundant cardiac annexins, until annexin A4 (A4) was identified both in the normal heart tissue and in cardiomyocytes [21, 22, 23, 24].

In pathology, the pattern of annexin up‐regulation appeared isoform‐specific and related to the severity of the cardiac disease. For example, A4 up‐regulation was reported in ventricles of human hearts with ischemic or dilated cardiomyopathy [23], inflammatory myopathies [24], in patients undergoing heart transplantation for heart failure, and in mice with myocardial ischemia/reperfusion injury [22, 24]. A4 overexpression in murine hearts using AAV9 as a vector showed that A4 prevents NFκB translocation in the ischemia/reperfusion injury model [22]. Additionally, annexins A2 and A5 were reported upregulated in patients with end‐stage heart failure [23, 25] or hypertensive heart disease [26]. The upregulation and redistribution of A5 to intracellular structures was accompanied by contractility reduction [26]. Annexin A6 upregulation was transient during the transition from compensatory hypertrophy to heart failure [27], followed by downregulation in the heart failure stage [25]. Activated A6 colocalized with atrial natriuretic peptide secretion granules to the membrane to facilitate its secretion [28]. Moreover, A6 overexpression was linked to reduced intracellular free Ca^2+^ both in diastole and systole and to accelerated Ca^2+^ uptake [10] possibly due to the association with SR membrane where it regulates the Ca^2+^ release/uptake cycle [29]. To date, these reports highlight the important role of annexins as modulators of calcium‐dependent mechanisms either via the calcium buffering capacity or as membrane adaptors, albeit with isoform‐specific effects on cardiac pathology.

Adenylyl cyclases (AC) AC5 and AC6 are the major cardiac AC isoforms. Upon βAR and Gαs activation, AC increases cAMP production. As a key enzyme in the βAR signaling pathway, mice with AC5 overexpression displayed exacerbated catecholamine‐induced cardiac remodeling such as greater left ventricular dilation and increased fibrosis, apoptosis, and hypertrophy [30, 31], while the inactivation of AC5 protected against catecholamine‐mediated or pressure‐induced effects in the heart and showed protection from aging‐induced cardiomyopathy [32, 33, 34]. While these studies consistently supported that most of the detrimental functional remodeling induced by βAR was mediated by AC5, the role of AC6 may be linked to protective effects as suggested by heart‐directed expression of AC6 in different models of cardiac stress [35] including pressure overload [36, 37]. Thus, the treatment of cardiovascular diseases may benefit from a specific and selective inhibition or activation of AC5 or AC6, respectively [38].

Our previous studies identified AC as a molecular target of annexin A4 [21]. Annexin A4 has been proven to negatively modulate specifically AC type 5 (AC5), but not AC6, via the N‐terminal region [39]. In A4‐deficient mice, the lack of interaction between A4 and AC5 has proven to be important for the cAMP dependent signal transduction under acute βAR stimulation, leading to the prolongation of action potential duration due to the increase of L‐type calcium current [39].

The role of A4 in cardiac pathophysiology remains poorly understood. The present study aimed to further explore at the cellular level the impact of A4 deficiency on the electrical remodeling induced by chronic βAR stimulation. Our data demonstrate that ventricular myocytes isolated from A4 deficient mice displayed advanced hypertrophy and altered current signature. These findings suggest a protective role for annexin A4 in the progression of sympathetic induced hypertrophy.

## 2 Materials and Methods

All experiments involving commercially available products were performed according to the manufacturer's specifications. This section is detailed in the Supporting Information.

### 2.1 Experimental Animals

FVB/N mice deficient in annexin A4 (anxa4a^−/−^) and the corresponding phenotype were previously described [40]. Male anxa4a^−/−^ (GT, gene trapped) and wild type anxa4a^+/+^ (WT) littermates were used at 19–23 weeks of age. All procedures were conducted in accordance with local animal welfare authorities and approved by the Landesamt für Natur, Umwelt und Verbraucherschutz Nordrhein Westfalen, Germany (Ref. no. 84_02.04.2015.A504).

### 2.2 Chronic Isoproterenol Treatment

Osmotic mini‐pumps (model 2001, Alzet, Cupertino, CA, USA) were implanted for 7 days into GT and WT mice for continuous application of 30 mg/kg body weight/day of isoproterenol (ISO) (DL‐isoproterenol hydrochloride, Merck, Darmstadt, Germany) dissolved in a solution containing 2 mM HCl in 0.9% NaCl. ISO‐treated mice were labeled GT_ISO_ and WT_ISO_. The control animals, NaCl‐treated groups: GT_NaCl_ and WT_NaCl_, respectively, received osmotic mini‐pumps filled with 2 mM HCl in 0.9% NaCl. Animals of different genotypes were randomly assigned to receive either NaCl or ISO treatment.

### 2.3 Heart Size

Mice were euthanized with CO_2_ and hearts were excised, cleaned from tissue remnants, washed with 0.9% NaCl solution, and patted dry. The hearts were weighed and normalized to the corresponding tibia length.

### 2.4 Isolation of Adult Mouse Ventricular Cardiomyocytes

Cardiomyocytes were isolated from adult mice from all four experimental groups (WT_NaCl_, GT_NaCl_, WT_ISO_, GT_ISO_) by enzymatic digestion [21]. The cells were separated by mechanical dispersion and Ca^2+^ concentration progressively increased to 1 mM before initiating the experiments.

### 2.5 Cell Size Measurements

Isolated ventricular myocytes were imaged using an inverted microscope (Nikon Eclipse Ti S, Nikon Instruments Inc., Tokio, Japan). Cell length, width, and area were measured from a minimum of *n* = 50 cardiomyocytes per mouse, for *N* = 4 mice from each experimental group. Averages per mouse of the cell parameters were used for statistical comparison between groups.

### 2.6 Fibrosis Masson Goldner Staining

Heart slices were stained using the Masson‐Goldner method according to the manufacturer's protocol. The collagen fibers were stained blue. Adjacent images from the same slice were taken using a Nikon Eclipse Ti S microscope and were stitched to single pictures using NIS‐Elements BR Analysis 4.13.05 Software. Image‐Pro Analyzer V 7.0 software was used to assess the fibrotic area expressed as a percentage of the number of blue pixels of the total number of pixels.

### 2.7 Western Blot

Ventricles were homogenized in lysis buffer supplemented with phosphatase and protease inhibitor tablets (A32959, ThermoFisher Scientific, Germany). Forty μicrogram of protein of each sample were equally distributed and loaded on 10% SDS‐PAGE gels. Equal protein load was assessed after transfer using Ponceau stain. The primary and secondary antibodies used for these experiments are presented in the Supporting Information. The bands were developed with SERVALight Helios (42587.03, SERVA Electrophoresis GmbH, Germany). Band intensities were quantified using ImageJ 1.49v (RRID:SCR_003070; NIH, Bethesda, MD, USA), normalized to Ponceau for load, and then to the average protein expression in WT_NaCl_.

### 2.8 qRT‐PCR

Total RNA was isolated from cardiac ventricles of mice from the four experimental groups using the TRIzol reagent (Life Technologies, Darmstadt, Germany). Total RNA (1 μg) was reversely transcribed to cDNA using the first strand cDNA synthesis kit for qRT‐PCR AMV (Roche Diagnostics, Cologne, Germany). Detection was performed with the LightCycler 1.5 System (Roche Diagnostics, Cologne, Germany) as described before [21]. The mouse primers used for these experiments are provided in the Supporting Information. Relative gene expression was calculated based on the $2^{-∆∆C_{T}}$ with the software tool REST, ver. 2.013 [41], using *Gapdh* as housekeeping gene and normalized further to WT_NaCl_.

### 2.9 Cellular Electrophysiology

Ca^2+^ tolerant ventricular myocytes were used for the measurements of action potentials and ionic currents (*I*_K_, *I*_CaL_, *I*_NCX_), as previously described [42, 43, 44] using amphotericin B perforated whole cell patch clamp method. Experiments were performed at room temperature using an EPC 800 amplifier (HEKA Elektronik, Lambrecht, Germany). Series resistance and membrane capacitance were compensated before data acquisition. The myocytes were kept in 1 mM CaCl_2_ normal bath solution at room temperature and were recorded within 5 h after isolation. Only quiescent myocytes presenting clear striations and rectangular shape were used for recordings. The detailed description of the protocols, solutions, and data analysis are provided in the Supporting Information. The investigator was blinded to the group allocation both during recording and analysis of the study.

### 2.10 Ca Transients and Sarcomere Length Shortening

Calcium transients (CaT) and sarcomere length (SL) were measured simultaneously in single cardiomyocytes using a Nikon Eclipse Ti‐S microscope, a MyoCam‐S camera (IonOptix, USA), and IonWizard 6.4.1.73 software. Cells were loaded with the ratiometric dye Indo‐1 AM (Invitrogen) for 10 min in the dark in the presence of Pluronic to enhance dye uptake, followed by 5 min for de‐esterification. Indo‐1 exhibits fluorescence shift depending on calcium binding (~400 nm bound, ~475 nm unbound) upon excitation at ~340 nm, reflecting intracellular calcium levels. Electrical field stimulation (2 ms duration, 20 V, 0.5 Hz) was applied for 10 min using a MyoPacer Cell Stimulator (IonOptix) before measurement. Baseline calcium transients and sarcomere shortening were recorded from 10 to 12 cells under basal conditions and after acute isoproterenol (1 μM) stimulation. The ratio of fluorescence emission (405 nm/495 nm) indicates the variation of the cytosolic Ca^2+^ concentration and was analyzed for baseline, amplitude, and kinetics of Ca^2+^ uptake. SL changes were analyzed using fast Fourier transform‐based software to estimate the fractional shortening (%FS) as percentage of diastolic SL length. Cell boundaries were manually adjusted to minimize background fluorescence. Data analysis was performed with IonWizard 6.4.1.73.

### 2.11 cAMP Measurements

Cardiomyocytes from each experimental group were treated with isoproterenol (1μM) or vehicle (water) in PBS for 5 min at 37°C and in 5% CO_2_, as previously described [39]. The cells were lysed and the supernatant containing the cytosolic fraction was used for cAMP measurements using the Amersham cAMP Biotrak enzyme immunoassay (ELISA, GE Healthcare, Little Chalfont, United Kingdom), starting from an input volume corresponding to 0.3 μg protein. The optical density was detected at 450 nm within 30 min using a Mithras LB 940 Microplate Analyzer (Berthold Technologies, Bad Wildbad, Germany). After subtracting the optical density (OD) corresponding to the non‐specific binding, the sample OD was normalized to the OD of the zero standard and extrapolated from the cAMP standard curve to derive the cAMP concentration. For each experimental design, the cAMP levels were expressed as cAMP quantity normalized to the total protein content of the input volume.

### 2.12 ECG Measurements

ECG measurements were performed in isoflurane‐anesthetized mice. Electrocardiograms were acquired from 7 to 8 mice for each of the four experimental groups, as previously described (Heinick et al. [39]). Briefly, the mice were anesthetized by inhalation with isoflurane‐nitrous mixture, according to the approved animal protocol. Mice were positioned supine on a 37°C heating plate and 5 subcutaneous limb electrodes were placed after loss of toe pinch reflex. For electrocardiogram recordings, electrodes were connected over an external biological amplifier (Dual Bio Amp, ADInstruments, Dunedin, New Zealand) to a data acquisition unit (PowerLab 2/20, ADInstruments, Dunedin, New Zealand), which recorded lead I and II and calculated lead III using LabChart 7 Pro software (ADInstruments, Dunedin, New Zealand). The protocol included a 5 min recording phase under basal conditions followed by an additional 30 min recording phase after intraperitoneal injection of isoproterenol (2 mg/kg body weight). Lead II was used for the estimation of heart rate (HR), PR interval, and QT interval measured before and after acute ISO application.

### 2.13 Statistical Analysis

Data are presented as mean ± SEM or mean ± SD, as described in the figure legend, from *n* cells and *N* mice. The normal distribution was tested using the Shapiro–Wilk normality test. Two‐tailed unpaired parametric or nonparametric *t*‐test (Mann–Whitney) was used for two group comparisons, according to the Gaussian distribution. For multi group comparison, one‐way ANOVA or two‐way repeated measures (RM) ANOVA was followed by Holm‐Sidak post hoc test. The sample size for the single cell measurements was selected to yield 80% statistical power at the significance level of *α* = 0.05. GraphPad Prism version 8.0 (RRID:SCR_002798; GraphPad Software, La Jolla California, USA) and SigmaPlot 11.2 (RRID:SCR_003210; Systat Software, San Jose, CA) were used for statistical analysis. Data were considered statistically different at *p* < 0.05.

## 3 Results

### 3.1 Chronic β‐Adrenergic Stimulation‐Induced Hypertrophy of GT Myocytes

**FIGURE 1:**
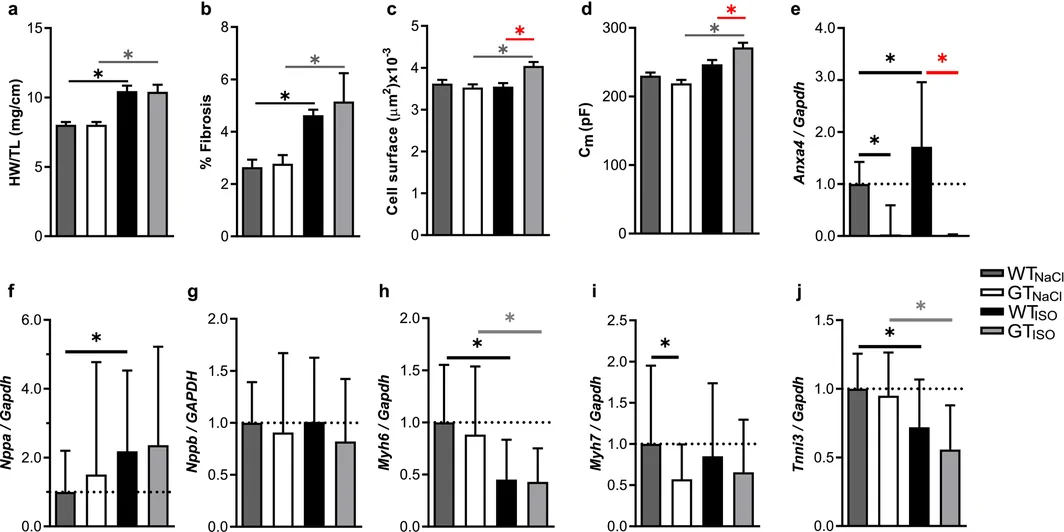
The effect of chronic ISO stimulation at structural and molecular level. (a) Heart size, (b) fibrosis, (c) cell surface area, (d) membrane capacitance (Cm), and mRNA levels of (e) *Anxa4*, (f) *Nppa*, (g) *Nppb*, (h) *Myh6*, (i) *Myh7*, and (j) *Tnni3*. Bar graphs show mean ± SEM. The legend of bar graphs is the same for all panels. *N* = 5–6 mice (a–c), *n* = 113–150 cells of *N* = 13–17 mice (d) and *N* = 9 mice per group (e–j); *p < 0.05 (as shown), where black was used to compare WT groups, gray for GT groups and red for ISO‐treated groups; one‐way ANOVA, Holm‐Sidak post‐test (a–d), and *t*‐test Rest 2.0 software statistical analysis (e–j), relative expression vs. *Gapdh*, and WT_NaCl_.

Chronic βAR stimulation induces ventricular hypertrophy by increasing fibrosis, cell size, or both. First, we assessed the changes at structural and transcriptional levels after chronic infusion of ISO in A4 deficient (GT_ISO_) and wild type (WT_ISO_) mice in comparison with NaCl‐treated time‐ and age‐matched control genotypes (WT_NaCl_ and WT_NaCl_, respectively). Within 7 days of chronic ISO infusion, no mortality event was detected in WT or GT mice. Figure 1 shows that heart hypertrophy and fibrosis increased to similar levels in both WT_ISO_ and GT_ISO_ mice versus NaCl‐treated mice, without a significant difference between ISO‐treated groups (Figure 1a,b). At the cellular level, the surface areas of ventricular myocytes (VM) were increased in GT_ISO_ versus both GT_NaCl_ and WT_ISO_ (Figure 1c), results confirmed by increased membrane capacitance (Cm) of the myocytes used in patch clamp recordings (Figure 1d).

At molecular level, the mRNA levels of *AnxA4* were moderately increased in WT_ISO_ and remained at low levels in GT_ISO_ hearts (Figure 1e). The analysis of the hypertrophy markers showed increased mRNA levels for the atrial natriuretic peptide (*Nppa*), decreased levels for myosin heavy chain 6 (*Myh6*, encoding alpha heavy chain subunit of cardiac myosin) and troponin I3 (*Tnni3*) with ISO treatment. *Nppa* did not increase in GT_ISO_ versus GT_NaCl_. No differences were detected between WT_ISO_ and GT_ISO_. The mRNA levels of natriuretic peptide B (*Nppb*) and myosin heavy chain 7 (*Myh7*, encoding beta heavy chain subunit of cardiac myosin) remained unchanged with ISO treatment in both genotypes (Figure 1f–j).

These results confirmed that chronic ISO treatment induced hypertrophy at the whole heart level with a moderate upregulation of *Anxa4* in WT mice. In the absence of A4, myocyte hypertrophy was detected in GT_ISO_ versus WT_ISO_.

### 3.2 Chronic β‐Adrenergic Stimulation Induced APD Prolongation in GT Myocytes

**FIGURE 2:**
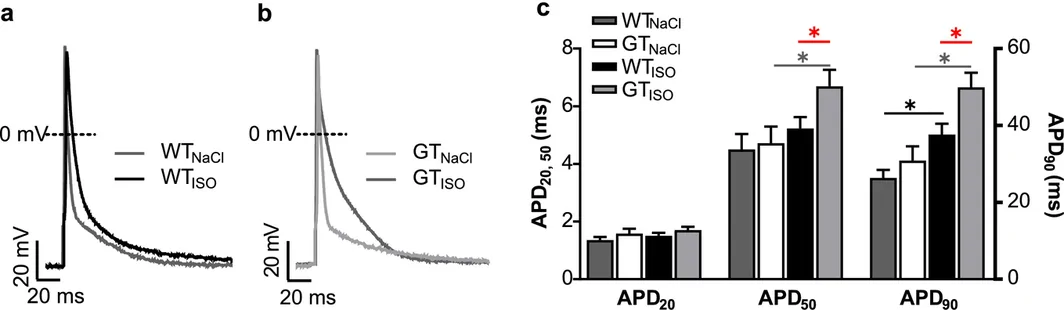
The effect of chronic ISO stimulation on AP duration. (a, b) Representative AP traces of ISO‐treated vs. NaCl‐treated WT (a) and GT (b) myocytes. (c) Bar graph shows mean ± SEM of the duration of AP at 20% (APD_20_), 50% (APD_50_) and 90% (APD_90_) repolarization, for *n*/*N*, *n* cells from *N* mice as follows 24/7 (WT_NaCl_), 21/6 (GT_NaCl_), 29/8 (WT_ISO_) and 39/9 (GT_ISO_). *p < 0.05 (as shown), where black was used to compare WT groups, gray for GT groups and red for ISO‐treated groups; one‐way ANOVA, Holm‐Sidak post hoc test.

Next, to test whether functional alterations occurred simultaneously with hypertrophy, we evaluated the electrical activity at baseline. Action potentials (AP) were recorded in VM isolated from the four experimental groups. Figure 2 shows two representative AP traces of ISO‐ versus NaCl‐treated WT (Figure 2a) and GT (Figure 2b) myocytes. The mean AP duration (APD) measured at 20%, 50%, and 90% repolarization (APD_20_, APD_50_, and APD_90_, respectively) showed a significant prolongation of APD_50_ by 42% only in GT_ISO_ versus GT_NaCl_, and increased APD_90_ by 43% in WT_ISO_ and by 62% in GT_ISO_, respectively, versus NaCl‐treated genotypes (*p* < 0.05, one‐way ANOVA) (Figure 2c). Chronic ISO‐treatment increased APD_50_ by 28% and APD_90_ by 33%, in GT_ISO_ versus WT_ISO_, respectively, thus indicating that more AP prolongation occurred at baseline in GT_ISO_ mice.

### 3.3 Molecular Basis of APD Prolongation in GT_ISO_ Mice

To determine whether APD prolongation was caused by a decrease of the outward K^+^‐current or an increase of inward Ca^2+^‐current, we performed voltage clamp recordings to assess the AP repolarization underlying currents.

#### 3.3.1 Potassium Currents in Chronically ISO‐Infused GT Myocytes

**FIGURE 3:**
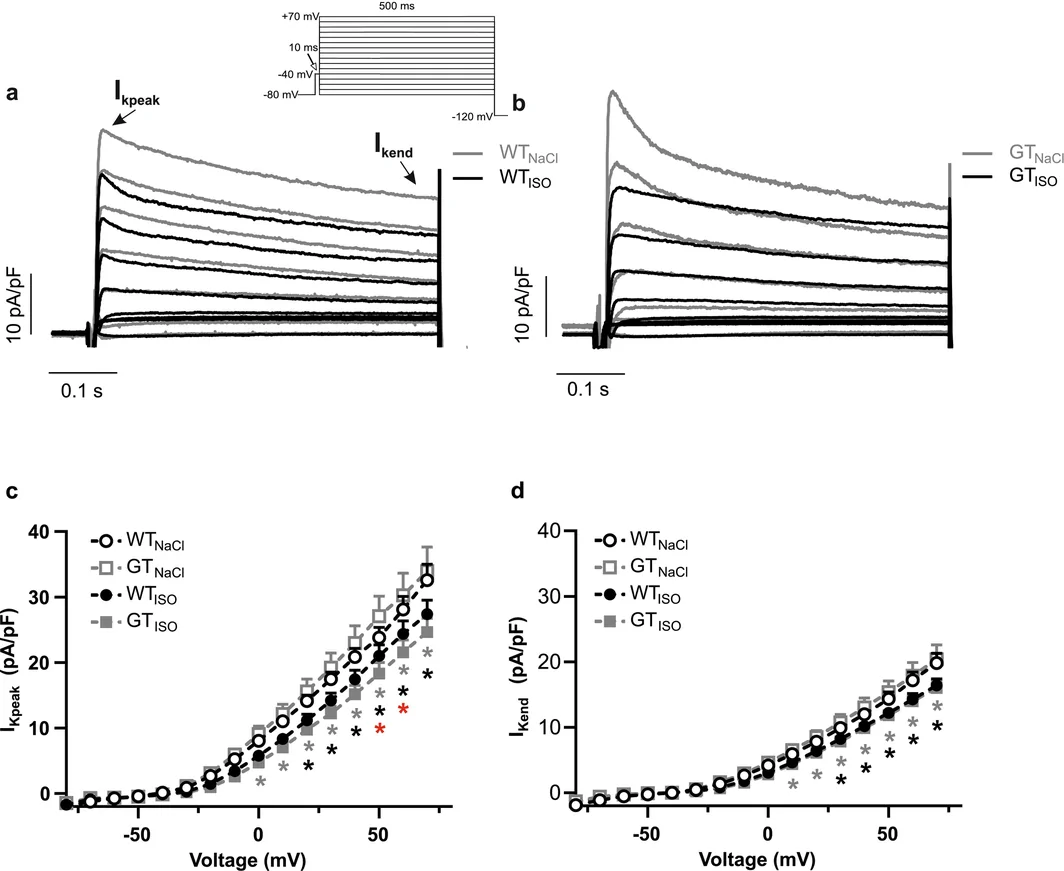
Chronic ISO stimulation decreased total outward K^+^‐currents. (a, b) Representative recordings of the total outward K^+^‐current in VM isolated from ISO‐ vs. NaCl‐treated WT (a) and GT (b) mice in response to the voltage protocol shown in the inset. For clarity, the traces are displayed with +20 mV increments from −80 mv to +60 mV. (c, d) I–V plots of total outward K^+^‐current density (pA/pF) measured at the beginning of the pulse (*I*_Kpeak_, c) or at the end of the pulse (*I*_Kend_, d). Data show mean ± SEM for *n*/*N*, *n* cells from *N* mice as follows 20/7 (WT_NaCl_), 16/6 (GT_NaCl_), 22/8 (WT_ISO_), and 20/9 (GT_ISO_). *p < 0.05 (as shown), where black was used to compare WT groups, gray for GT groups and red for ISO‐treated groups; two‐way RM ANOVA, Holm‐Sidak post hoc test.

Total outward currents were recorded using the voltage protocol shown in Figure 3a (inset). Figure 3a,b show paired representative traces recorded from myocytes isolated from the four experimental groups. Current traces measured at peak (*I*_Kpeak_) and at the end of the depolarizing pulse (*I*_Kend_) were normalized to the cell capacitance for quantification (Figure 3c,d). *I*_Kpeak_ and *I*_Kend_ were not different between GT_NaCl_ and WT_NaCl_. At all potentials above 0 mV, the average effect of chronic ISO treatment was a reduction of *I*_Kpeak_ and *I*_Kend_ in WT_ISO_ by 16% (*p* < 0.05, WT_ISO_ vs. WT_NaCl_), while in GT_ISO_ the *I*_Kpeak_ was reduced by 32% and *I*_Kend_ by 23% (*p* < 0.05, GT_ISO_ vs. GT_NaCl_). A moderate but significant 11% decrease of *I*_Kpeak_ was detected in GT_ISO_ versus WT_ISO_ (*p* < 0.05).

**FIGURE 4:**
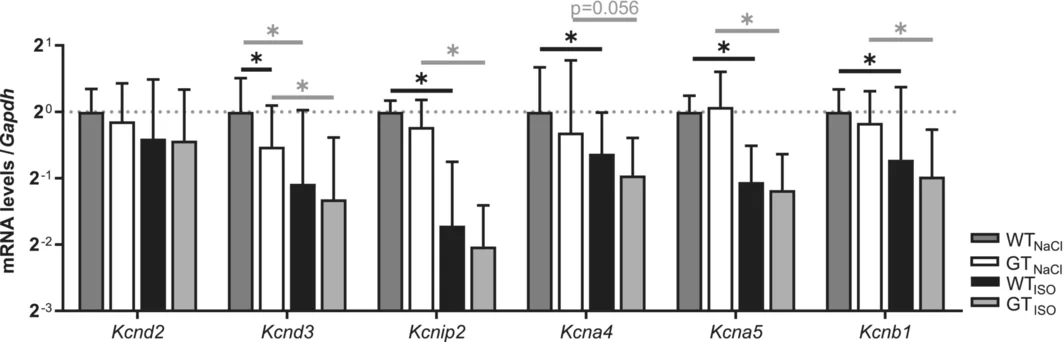
Chronic ISO stimulation decreased the mRNA level of many genes encoding K^+^ channel α‐subunits. mRNA levels were normalized to *Gapdh* and are presented as fold change relative to WT_NaCl_. Bar graphs show mean ± SEM (*N* = 9 per group). *Y*‐axis is represented on a log_2_ scale. *p < 0.05 (as shown), where black was used to compare WT groups, gray for GT groups; Rest 2.0 software statistical analysis.

Several K^+^‐channels contribute to the total outward K^+^‐current. To verify whether the decrease of *I*_K_ was accompanied by molecular changes at mRNA levels of the main K^+^‐channels underlying AP repolarization, we used qRT‐PCR to analyze the genes encoding the α‐subunits of the K^+^‐channels with fast kinetic of activation Kv4.2 (encoded by *Kcnd2*), Kv4.3 (encoded by *Kcnd3*), Kv1.4 (encoded by *Kcna4*), and Kv1.5 (encoded by *Kcna5*), which likely contribute predominantly to *I*_Kpeak_. Among the K^+^‐channels contributing to *I*_Kend_, we evaluated K^+^‐channels with slow kinetic of inactivation, such as Kv1.5 and Kv2.1 (encoded by *Kcnb1*). Moreover, we assessed the mRNA level of *Kcnip2* encoding for the β‐auxiliary subunit Kv4.x channel‐interacting protein 2 (KChIP2), a subunit with a known role in channel trafficking to the membrane [45]. Figure 4 shows the fold change relative to WT_NaCl_ of the mRNA levels normalized to *Gapdh* from all four experimental groups. There were no major differences in mRNA levels in GT_NaCl_ versus WT_NaCl_, except for a 30% decrease of *Kcnd3* levels (*p* < 0.05). Irrespective of genotype, mRNA levels of *Kcnd3*, *Kcnip2*, *Kcna4*, *Kcna5*, and *Kcnb1* decreased to similar levels due to chronic ISO infusion versus the corresponding NaCl‐treated control.

**FIGURE 5:**
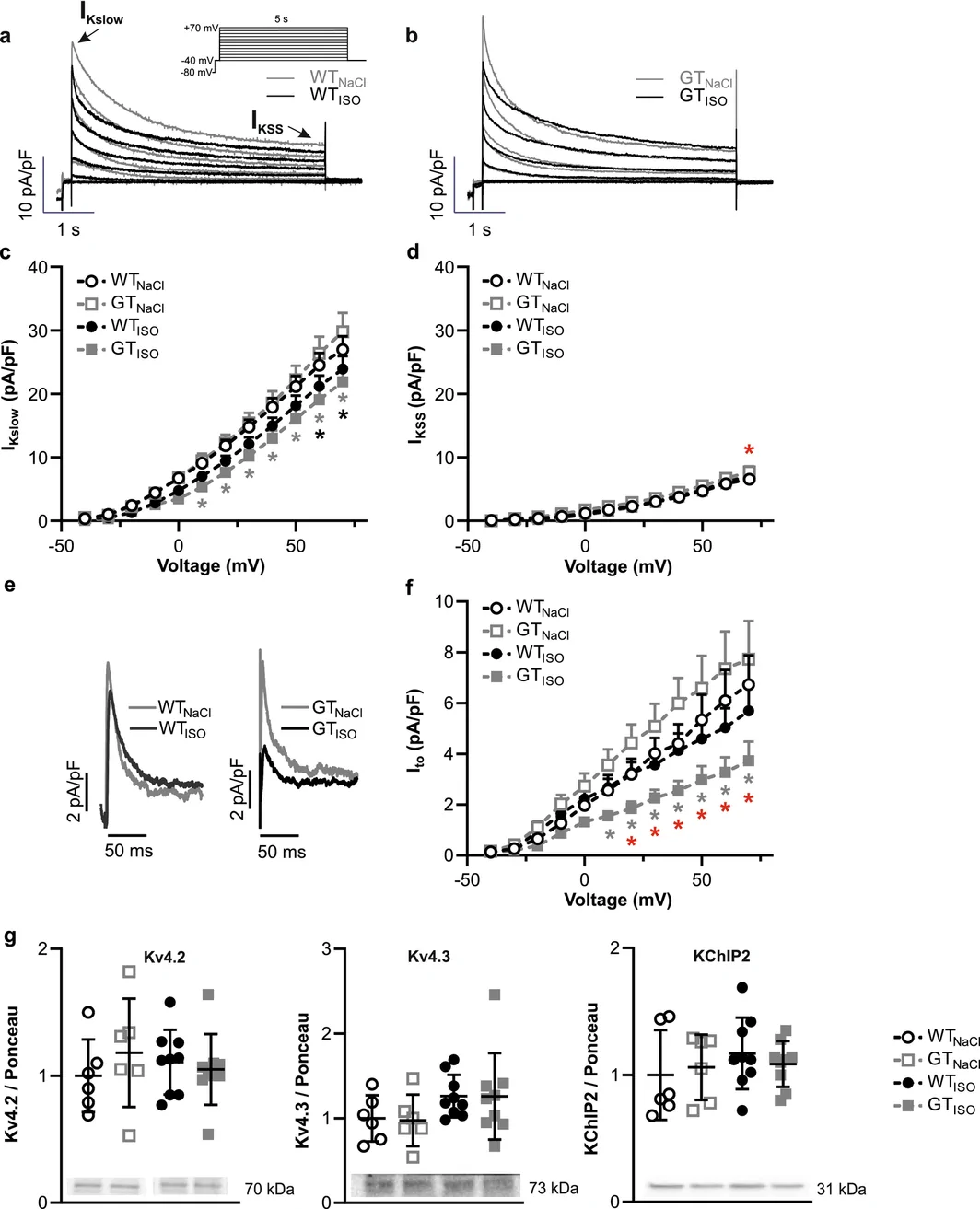
Chronic ISO stimulation reduces the transient outward K^+^‐current (I_to_) in GT more than in WT. (a, b) Representative traces of the K^+^‐current recorded in VM isolated from ISO‐treated vs. NaCl‐treated WT (a) and GT (b) mice, using the voltage protocol shown in the inset. Panels a, and b show traces from −40 to +60 mV with +20 mV increments. (c, d) I–V plots of the K^+^ current density measured at the beginning of the pulse or at peak (*I*_Kslow_, c) or at the end of the pulse (*I*_Kss_, d). (e) Representative traces of the subtracted *I*_to_ current elicited at +40 mV in VM isolated from WT_ISO_ (left) and GT_ISO_ (right) mice vs. corresponding NaCl‐treated controls. (f) I–V plots of the *I*_to_ current density. Data show mean ± SEM of corresponding current densities. (c, d, f) of *n*/*N*, *n* cell isolated from *N* mice as follows 14/7 (WT_NaCl_), 14/6 (GT_NaCl_), 20/8 (WT_ISO_), and 18/9 (GT_ISO_); (g) Scattered plots show mean ± SD of the protein levels normalized to Ponceau and calculated relative to WT_NaCl_. Representative blots showing the protein expression of Kv4.2, Kv4.3, KChIP2 in the 4 groups are presented in the inset. The number of mice *N* was between 5 and 9 per group. *p < 0.05 (as shown), where black was used to compare WT groups, gray for GT groups and red for ISO‐treated groups; two‐way RM ANOVA (c, d, f) and one‐way ANOVA, Holm‐Sidak post hoc test (g).

Two current components contribute to the *I*_Kpeak_: (1) a fast activating and fast inactivating transient outward component, *I*_to_, and (2) a slow K^+^‐current, *I*_Kslow_, with a fast kinetic of activation and a slow kinetic of inactivation. These two components were separated using a voltage prepulse of 100 ms duration to −40 mV to inactivate Kv4.x channels, as in the voltage protocol shown in the inset of Figure 5a. Representative traces recorded using this protocol are shown for WT (Figure 5a) and GT (Figure 5b) experimental groups. Peak (*I*_Kslow_) and steady‐state (*I*_Kss_) outward current densities plotted versus voltage (I‐V plots) showed that due to chronic ISO treatment, *I*_Kslow_ declined on average at all potentials above 0 mV by 16% in WT_ISO_ (*p* < 0.05, WT_ISO_ vs. WT_NaCl_) and by 31% in GT_ISO_ (*p* < 0.05, GT_ISO_ vs. GT_NaCl_), with no difference between GT_ISO_ and WT_ISO_ (Figure 5c). No significant differences were detected for the *I*_Kss_ current density due to ISO‐treatment of either genotype (Figure 5d).

Next, *I*_to_ currents were assessed as the −40 mV sensitive current by subtracting traces recorded with the protocols of Figures 3 and 5a. Representative *I*_to_ currents are shown in Figure 5e and the corresponding I‐V plots in Figure 5f. Due to ISO treatment, *I*_to_ declined on average at all potentials above 0 mV, by 13% in WT_ISO_ vs. WT_NaCl_ (N.S.), by 56% in GT_ISO_ versus GT_NaCl_ (*p* < 0.05), and by 37% in GT_ISO_ versus WT_ISO_ (*p* < 0.05). These results were in line with the reduced mRNA levels of *Kcnd3* and *Kcnip2* in ISO‐ versus NaCl‐treated groups (Figure 4). To test whether the decrease of current is a consequence of decreased protein expression, Western blot assay for the α‐subunits of Kv4.2, Kv4.3, and of the β‐subunit KChIP2 were evaluated using Ponceau as loading control (representative blots in Figure 5g). Quantitative analysis showed no difference among groups in the expression of these proteins (Figure 5g).

#### 3.3.2 L‐Type Calcium Current in Chronically ISO‐Infused GT Myocytes

**FIGURE 6:**
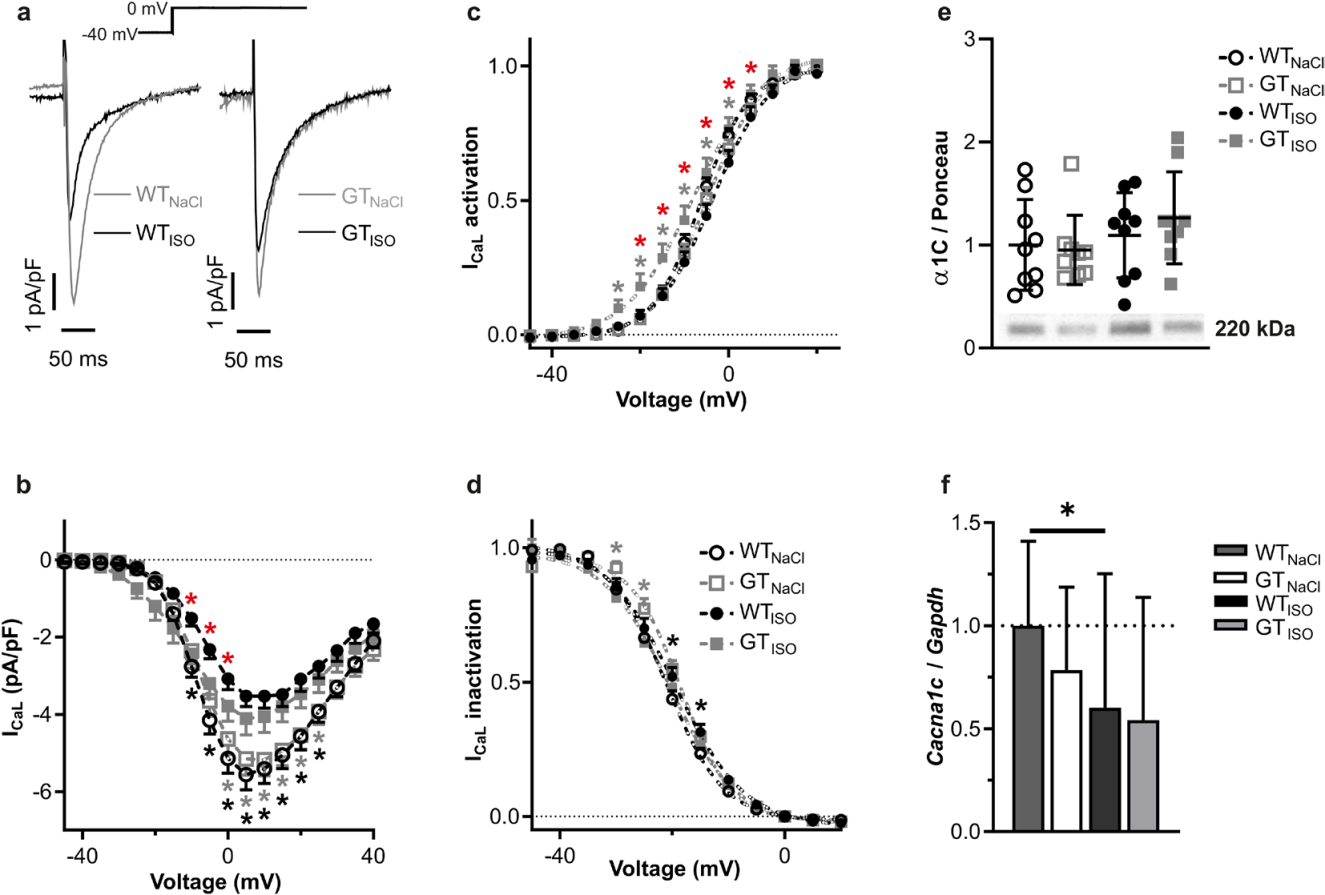
The effect of chronic ISO stimulation on L‐type calcium channel and current (*I*_CaL_). (a) Representative traces of *I*_CaL_ elicited by a depolarizing pulse to 0 mV from −40 mV in ISO‐treated vs. NaCl‐treated WT (left) and GT (right) myocytes. (b) I–V plots of *I*_CaL_ in the four groups; (c) Voltage dependence of activation; (d) Voltage dependence of inactivation. Data show mean ± SEM (b–d) of *n*/*N*, *n* cells isolated from *N* mice as follows 18/7 (WT_NaCl_), 17/9 (GT_NaCl_), 18/7 (WT_ISO_), and 13/7 (GT_ISO_). The legend for panels (b–d) is the same and displayed in panel (d). (e) Scattered plots showing mean ± SD of the α‐subunit of L‐type calcium channel (α1C) protein levels normalized to Ponceau. Results are presented as fold change relative to WT_NaCl_. Representative blots showing the protein expression of α1C are displayed in the inset. The number of mice *N* was between 7 and 9 per group (f) mRNA levels of *Cacna1c* were normalized to *Gapdh* and presented as fold change relative to WT_NaCl_. *Y*‐axis is represented on a log_2_ scale. Data show mean ± SEM for *N* = 9 mice per group. *p < 0.05 (as shown), where black was used to compare WT groups, gray for GT groups and red for ISO‐treated groups; two‐way RM ANOVA (b–d), one‐way ANOVA (e); Holm‐Sidak post hoc test (b–e), Rest 2.0 software statistical analysis (f).

Next, we analyzed the L‐type calcium current (*I*_CaL_) as the second major current underlying AP repolarization. Figure 6a shows representative *I*_CaL_ traces induced at 0 mV from a −40 mV holding potential from ISO‐ versus NaCl‐treated mice, WT and GT experimental groups, respectively. The I‐V plots for *I*_CaL_ showed that chronic ISO treatment induced an average reduction of *I*_CaL_ at 0 mV by 40% in WT_ISO_ versus WT_NaCl_ (*p* < 0.05) and by 18% in GT_ISO_ versus GT_NaCl_ (*p* < 0.05) (Figure 6b). The Ca^2+^ currents recorded in GT_ISO_ myocytes were 23% larger than in WT_ISO_ myocytes at −10 and 0 mV (*p* < 0.05). The voltage at 50% maximum activation (E50a) was shifted by −2.9 mV in GT_ISO_ versus GT_NaCl_ (N.S.) and by −4.2 mV in GT_ISO_ versus WT_ISO_ (*p* < 0.05), while in WT_ISO_ versus WT_NaCl_ E50a was shifted by +2.7 mV (*p* < 0.05) (Figure 6c). No changes were detected in the voltage dependence of inactivation (Figure 6d). Consequently, *I*_CaL_ displayed a 2‐fold increase of the integrated area of the window current in GT_ISO_ versus WT_ISO_ (WT_NaCl_: 0.53 ± 0.03, *n*/*N* = 18/7; GT_NaCl_: 0.71 ± 0.13, *n*/*N* = 17/9; WT_ISO_ 0.77 ± 0.21, *n*/*N* = 18/7 and GT_ISO_: 1.53 ± 0.36*^,#^, *n*/*N* = 13/7, *^,#^ *p* < 0.05 GT_ISO_ versus GT_NaCl_ (*) and WT_ISO_ (^#^), one‐way ANOVA Holm‐Sidak post hoc test) (Figure S2).

Western blot analysis showed that the protein levels of the α‐subunit of L‐type calcium channel (α1C) were not different between experimental groups (Figure 6e). The mRNA levels analysis confirmed that the *Cacna1c* levels were not different between GT_NaCl_ and WT_NaCl_. Following ISO treatment, *Cacna1c* mRNA levels were decreased in WT_ISO_ versus WT_NaCl_, and not in GT_ISO_ versus GT_NaCl_ (Figure 6f).

#### 3.3.3 Na^+^/Ca^2+^ Exchanger in Chronically ISO‐Infused GT Myocytes

**FIGURE 7:**
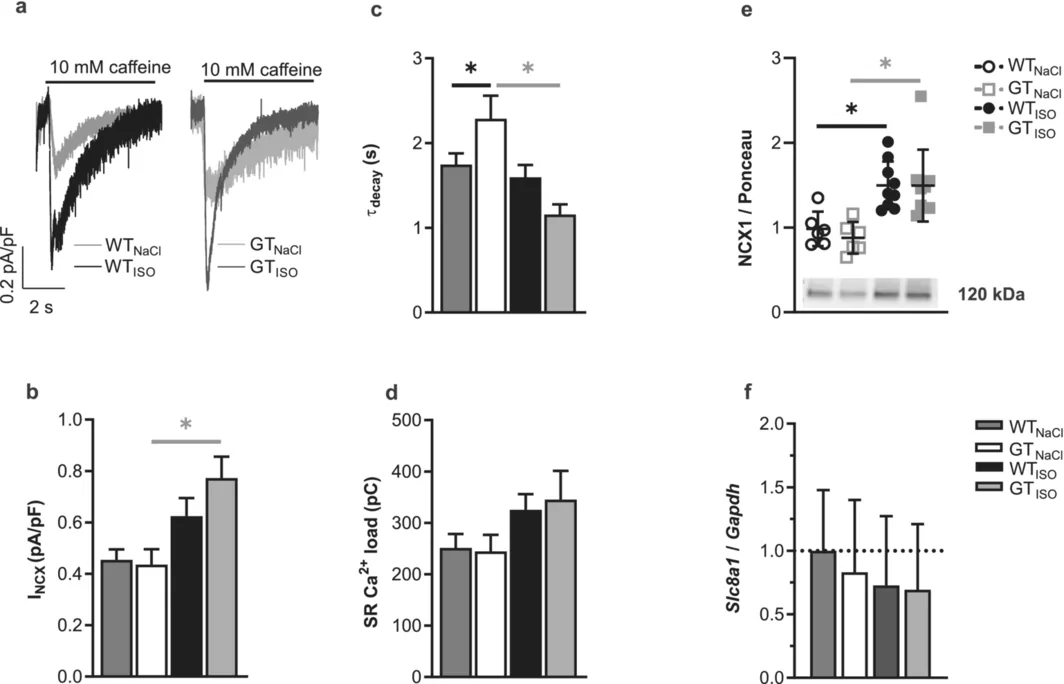
The effect of chronic ISO stimulation on Na^+^/Ca^2+^ exchanger (NCX1). (a) Representative traces of 10 mM caffeine‐induced current in ISO‐treated vs. NaCl‐treated WT (left) and GT (right) myocytes. (b) *I*_NCX_ current density, (c) exponential time constant of the current decay (τ_decay_), and (d) area under the curve as an index of sarcoplasmic reticulum Ca^2+^ load (SR Ca^2+^ load). Data show mean ± SEM (b–d) of *n*/*N*, *n* cells isolated from *N* mice as follows 9/4 (WT_NaCl_), 9/4 (GT_NaCl_), 9/5 (WT_ISO_) and 8/4 (GT_ISO_). The legend for panels (b–d) is the same and displayed in panel (d). (e) Scattered plots showing mean ± SD of the NCX1 protein levels normalized to Ponceau. Results are presented as fold change relative to WT_NaCl_. Representative blots showing the protein expression of NCX1 are displayed in the inset. The number of mice *N* was between 6 and 9 per group. (f) mRNA levels of *Slc8a1* normalized to *Gapdh* and presented as fold ratio relative to WT_NaCl_. Data show mean ± SEM for *N* = 9 mice per group. *p < 0.05 (as shown), where black was used to compare WT groups, gray for GT groups; one‐way ANOVA, Holm‐Sidak post hoc test (b–e), Rest 2.0 software statistical analysis (f).

As an electrogenic transporter, Na^+^/Ca^2+^ exchanger (NCX1) contributes to the late phase of AP repolarization [46]. To assess whether the level of hypertrophy achieved after 7 days of ISO infusion altered NCX1 activity or expression in WT or GT genotypes, we evaluated NCX1 at current (*I*_NCX_), protein, and mRNA levels. *I*_NCX_ was induced with a pulse of 10 mM caffeine (Figure 7a). Statistical analysis of the peak current amplitude, the exponential time constant of decay (τ_decay_, as a measure of NCX1 activity), and the integrated area under the curve to estimate the Ca^2+^ content of the sarcoplasmic reticulum (SR Ca^2+^ load) are shown in Figure 7b–d. ISO‐treated mice showed increased *I*_NCX_ and SR Ca^2+^ load and accelerated kinetics of the current decay, independent of genotype. The changes for *I*_NCX_ and τ_decay_ were significant in GT_ISO_ versus GT_NaCl_ but did not reach significance for SR Ca^2+^ load (*p* = 0.068). *I*_NCX_ of WT_ISO_ myocytes showed a similar trend without reaching statistical significance (*p* = 0.067), while no significance was calculated for τ_decay_ (*p* = 0.557) or for SR Ca^2+^ load (*p* = 0.160) in WT_ISO_ versus WT_NaCl_. No significant differences in these parameters were detected between GT_ISO_ and WT_ISO_. These data suggested that A4 depletion may create a substrate for NCX1 activation in response to chronic sympathetic stimulation.

Western blot and qRT‐PCR results showed a significant increase in the protein levels of NCX1 by 50% and a moderate decrease in the mRNA levels of *Slc8a1* (N.S., *p* = 0.07), respectively, under ISO treatment; however, there were no significant differences between ISO‐treated genotypes (Figure 7e,f).

### 3.4 The Response of Myocyte From ISO—Infused Mice to Acute ISO Stimulation

**FIGURE 8:**
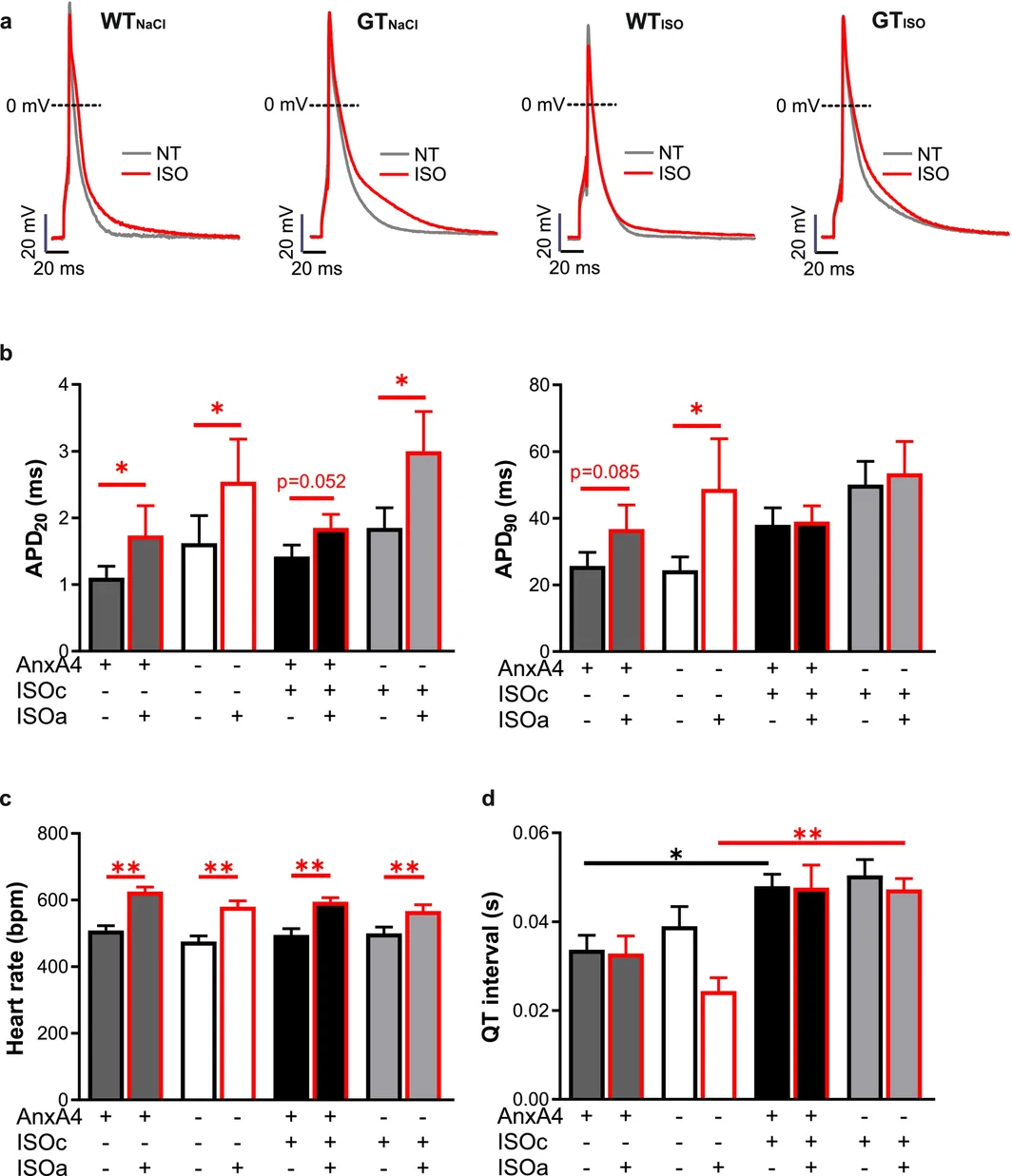
The effect of acute ISO stimulation on action potential duration and ECG after chronic ISO infusion. (a) Representative AP recorded before and after 1 μM ISO stimulation (red trace) in myocytes isolated from (left–right) WT_NaCl_, GT_NaCl_, WT_ISO_, and GT_ISO_. (b) Bar graphs show mean ± SEM of AP duration (APD) calculated at 20% (APD_20_) and 90% (APD_90_) repolarization (left to right) for *n* cells from *N* mice as follows 7/5 (WT_NaCl_), 7/4 (GT_NaCl_), 13/6 (WT_ISO_), and 10/5 (GT_ISO_). (c, d) Bar graphs show mean ± SEM of two parameters calculated from ECG recordings, before and after 1 μM ISO stimulation, heart rate expressed as beats per minute (bpm) (c) and duration of QT interval (d). *N* = 8 mice per group (c, d). “+” or “−” show the presence or the absence of A4, chronic ISO‐treatment (ISOc) and acute ISO stimulation (ISOa), respectively *p < 0.05 (as shown) one‐way ANOVA, Holm‐Sidak post hoc test.

To assess the degree of desensitization of the βAR after 7 days of ISO treatment, we recorded the AP in response to acute application of 1 μM ISO. Figure 8a shows representative traces of the AP before and after ISO application. The analysis of APD_20_ and APD_90_ showed that cardiomyocytes isolated from NaCl‐treated mice APD were prolonged by acute ISO stimulation at all levels in both WT and GT genotype, as it has been previously shown for untreated mice [39]. Acute ISO application increased APD_20_ above the baseline in both ISO‐treated groups to levels similar to NaCl‐treated myocytes while it had no effects at the APD_90_ level in chronically ISO‐infused mice, irrespective of genotype (Figure 8b). These results suggest that after 7 days of ISO infusion, the ion channels involved in APD prolongation at 90% repolarization, such as L‐type calcium channel, uncoupled from the regulatory mechanisms activated by βAR stimulation.

To test whether the alterations at cellular level have an impact on the whole heart function, ECGs have been recorded 10 min before and 30 min after ISO injection. Heart rates were not different at baseline and all hearts showed positive chronotropy in acute ISO (Figure 8c). For a direct comparison to single cell APD_90_ measurements, we analyzed the duration of ventricular repolarization, QT interval (Figure 8d). The results show that under basal conditions, QT interval was significantly longer in WT_ISO_ versus WT_NaCl_, and not significant in GT_ISO_ versus GT_NaCl_ (*p* = 0.1825) or WT_ISO_ (Figure S4a). QT interval duration was not changed with acute ISO stimulation in the four experimental groups (Figure 8d), as expected from an increase of APD_90_. QT interval duration was larger in acutely ISO‐stimulated GT_ISO_ versus acutely ISO‐stimulated GT_NaCl_ (*p* < 0.05). Two more parameters were measured from ECGs: the PR and QRS durations which were not different between groups in basal conditions. While PR did not change with acute ISO stimulation, QRS interval prolongation was longer in acutely ISO‐stimulated WT_ISO_ versus basal WT_ISO_ and versus acutely ISO‐stimulated GT_ISO_ (Figure S4b,c). Arrhythmia has been evaluated by measuring the number of ventricular extrasystoles (VES) during the 30 min of acute ISO application. The percentage of mice displaying VES was equal among the groups, with an average of 37.5%, irrespective of genotype or treatment. Two GT_NaCl_ mice displayed 4 and 5 VES, and one WT_NaCl_ mouse displayed 6 VES, while WT_ISO_ and GT_ISO_ mice displayed only one or two VES after acute ISO injection. Overall, these data show that there were no major differences between genotypes or with chronic treatment in the measured ECG parameters or in the predisposition to ventricular arrhythmia nor at basal or under a stress test.

#### 3.4.1 Calcium Transients in Chronically ISO‐Infused GT Myocytes

**FIGURE 9:**
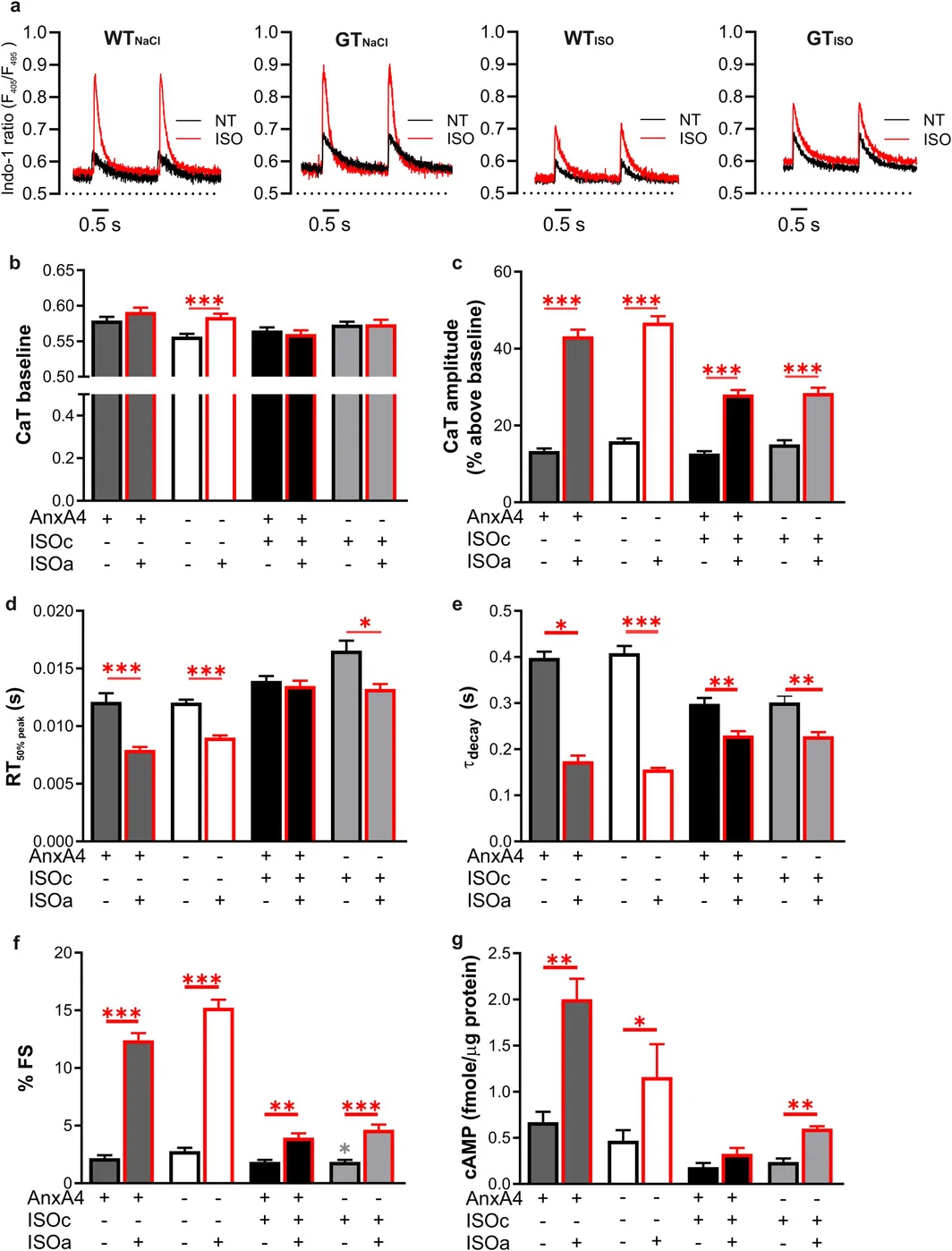
The effect of acute and chronic ISO stimulation on excitation‐contraction coupling. (a) Representative calcium transients' traces recorded before and after 1 μM ISO stimulation (red trace) in myocytes isolated from (left to right) WT_NaCl_, GT_NaCl_, WT_ISO_, and GT_ISO_. (b–f) Bar graphs show [Ca^2+^]_i_ at baseline (b), calcium transient (CaT) amplitude expressed as percentage of increase above the baseline (c), rise time to 50% from baseline to peak, RT_50%peak_, (d), the kinetic of Ca^2+^ uptake expressed as time constant (τ) of decay (e) and contractility expressed as sarcomere length fractional shortening (%FS, f). Data show mean ± SEM of *n*/*N*, *n* cells isolated from *N* mice as follows 50/5 (WT_NaCl_), 50/5 (GT_NaCl_), 60/6 (WT_ISO_), and 70/7 (GT_ISO_). (g) Bar graph showing global cAMP production in cardiomyocyte lysate before and after acute ISO stimulation. Data show mean ± SEM of cell lysates from *N* mice as follows 7 (WT_NaCl_), 7 (GT_NaCl_), 8 (WT_ISO_), and 8 (GT_ISO_). “+” or “−” show the presence or the absence of A4, chronic ISO‐treatment (ISOc) and acute ISO stimulation (ISOa), respectively. *p < 0.05 (as shown) one‐way ANOVA, Holm‐Sidak post hoc test.

Calcium transients (CaT) and sarcomere shortening were measured simultaneously to assess intracellular calcium and contraction in electrically stimulated isolated cardiomyocytes. Figure 9a shows representative traces of the CaT before and after acute ISO application in myocytes. Before ISO application, baseline [Ca^2+^]_i_ levels were smaller in GT_NaCl_ versus both WT_NaCl_ and GT_ISO_ (*p* < 0.05) (Figure S5a); CaT amplitudes were not different (Figure S5b); rise time to 50% peak (RT_50%peak_) and Ca^2+^ re‐uptake kinetics were significantly slower in chronically ISO‐ versus NaCl‐treated groups (*p* < 0.0001 both) (Figure S5c,d), while %FS was larger in GT_NaCl_ versus GT_ISO_ (*p* < 0.05) (Figure S5e).

Acute ISO stimulation significantly increased baseline [Ca^2+^]_i_ in GT_NaCl_ (Figure 9b). All groups responded to acute ISO with a significant increase in CaT amplitude; however, there was a larger response in WT_NaCl_ and GT_NaCl_, versus WT_ISO_ and GT_ISO_ (Figure 9c). Except for WT_ISO_, the cells responded to acute ISO with a significant decrease in RT_50%peak_ (Figure 9d), and all groups responded to acute ISO with a significantly faster Ca^2+^ uptake kinetic (Figure 9e) and an increase in contraction (Figure 9f). For all parameters, the changes induced by acute ISO were significantly reduced in chronically ISO‐ versus NaCl‐treated groups (*p* < 0.001), and no differences were detected between GT_ISO_ and WT_ISO_.

Many of these changes are mediated by PKA phosphorylation of different targets, such as phospholamban (PLB), ryanodine receptor, and L‐type calcium channel. PKA activation depends on cAMP levels and production, which were measured in the four groups, in the absence and presence of acute ISO, respectively. Under basal conditions, cAMP production decreased significantly in WT_ISO_ versus WT_NaCl_ (*p* < 0.05), and not in GT_ISO_ versus GT_NaCl_ (*p* = 0.588) myocytes (Figure S5f); however, in chronically ISO‐treated myocytes we measured lower cAMP levels than in NaCl‐treated myocytes. Despite the decrease of diastolic cAMP levels in ISO‐treated groups, PLB and CREB levels of phosphorylation were not different between genotypes or due to chronic treatment (Figure S5g,h).

cAMP produced upon acute ISO stimulation induced a significant increase of cAMP in WT_NaCl_, GT_NaCl_, and GT_ISO_ groups, while the increase in WT_ISO_ did not reach significance (*p* = 0.1605) (Figure 9g). The measurements of cAMP production are a readout of AC activity in these conditions.

These results suggest that chronically ISO‐infused myocytes retain the βAR sensitivity and are able to respond with positive lusitropy and inotropy, irrespective of genotype; however, with less intensity, indicating the presence of β‐adrenergic signaling desensitization.

**FIGURE 10:**
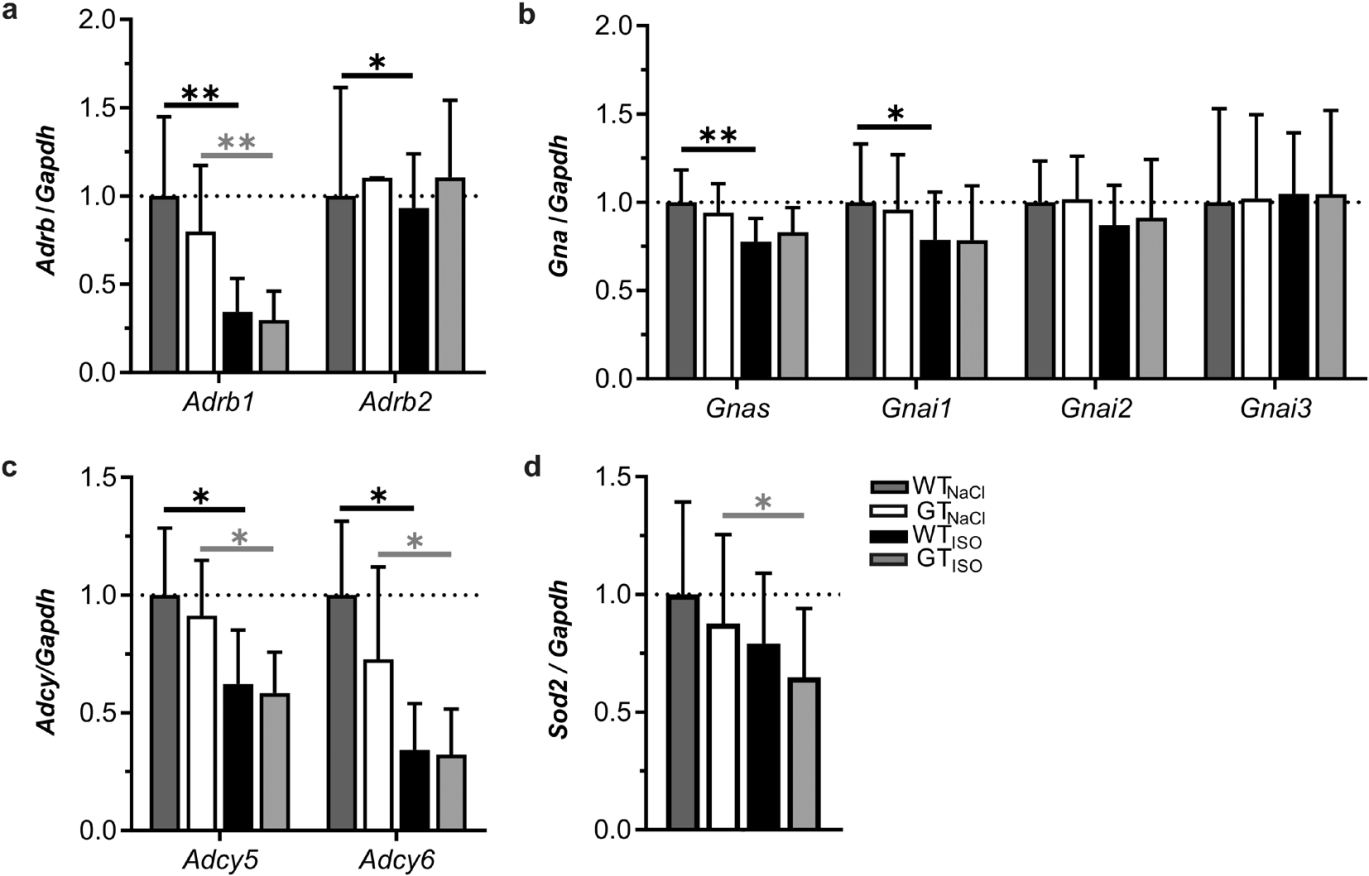
Chronic ISO stimulation alters mRNA level of β‐adrenergic signaling molecules. mRNA levels of (a) β‐adrenergic receptors 1 and 2 (*Adrb1/2*); (b) G alpha proteins (*Gnas/ix*) and (c) cardiac isoforms of adenylyl cyclases (*Adcy5/6*) and (d) Mn superoxide dismutase (*Sod2*) were normalized to *Gapdh* and presented as fold change relative to WT_NaCl_. Bar graphs show mean ± SEM (*N* = 9 per group). *Y*‐axis is represented on a log_2_ scale. *p < 0.05 (as shown), where black was used to compare WT groups, gray for GT groups; Rest 2.0 software statistical analysis.

To test if the desensitization of the β‐adrenergic signaling to chronic ISO infusion may have been caused by reduced expression of the main effector molecules involved in this pathway: β‐adrenergic receptors (βAR), G alpha (Gα) proteins and adenylyl cyclase isoforms, we measured the mRNA levels using qRT‐PCR. In WT_ISO_ versus WT_NaCl_, the mRNA levels of *Adrb1*, *Adrb2*, *Adcy5*, *Adcy6*, *Gnas* and *Gnai1* genes encoding for β1AR, β2AR, AC5 and AC6, Gαs and Gαi1, respectively, decreased significantly with ISO treatment (Figure 10a–c). In comparison, only Adrb1, Adcy5 and Adcy6 decreased in GT_ISO_ versus GT_NaCl_ suggesting a better preservation of the β2‐adrenergic signaling apparatus in GT_ISO_ versus GT_NaCl_ mice. No differences have been detected between GT_ISO_ and WT_ISO_, suggesting that the transcriptional program was influenced only by ISO and not by A4 deficiency. The decreases of AC5 and AC6 are in line with the reduced cAMP production (Figure 9g).

Animal models with AC5 overexpression displayed reduced protein expression of manganese superoxide dismutase (MnSOD or SOD2), thus displaying increased oxidative stress induced by chronic ISO [31]. To test whether the potential increase of AC5 activity due to the absence of A4, is associated with decreased antioxidant capacity in cardiomyocytes, we assessed the mRNA levels of *Sod2* (Figure 10d). Data show that chronic ISO treatment induced a decrease of *Sod2* mRNA levels in WT_ISO_ versus WT_NaCl_ (*p* = 0.06) and GT_ISO_ versus GT_NaCl_ (*p* < 0.05), and in GT_ISO_ versus WT_ISO_ (*p* = 0.078). These results support the likelihood that at this early level after chronic ISO infusion, A4 deficiency may create a molecular background for increased oxidative stress as compared to WT mice.

## 4 Discussion

In the current study, we used ISO‐infused annexin A4‐deficient mice (GT) to assess the contribution of annexin A4 (A4) to the electrical remodeling induced by chronic βAR stimulation. Our results showed that GT_ISO_ mice: (1) displayed myocyte hypertrophy, (2) increased AP duration likely due to an increased ratio of Ca^2+^ influx to K^+^ efflux, and (3) displayed increased *I*_NCX_ activity selectively in GT_ISO_ mice. All these changes were significantly diminished in WT_ISO_, at similar stages of the fetal gene program activation. Thus, our data showed advanced electrical and structural remodeling in GT_ISO_ at the cellular level and suggest that annexin A4, as an endogenous AC5 inhibitor, may play a protective role in the early phases of sympathetic hyperactivation, such as delaying the onset of myocyte hypertrophy and electrical remodeling.

### 4.1 Structural Remodeling

Chronic stimulation of βAR using ISO infusion is a well‐established experimental model used to recapitulate the onset of hypertrophy and progression to heart failure [47]. The role of β1AR overstimulation in cardiac hypertrophy and heart failure has been recently reviewed by Dhalla et al. [48], with strong evidence that the molecular mechanisms of remodeling are mediated both by protein kinase A and cAMP binding protein EPAC [49, 50]. Similar to studies using other mouse models, our results showed that GT_ISO_ mice presented increased heart size and fibrosis, hallmarks of cardiac remodeling associated with maladaptive hypertrophy [51]. At the stage of remodeling reached after 7 days of ISO infusion, no structural or molecular differences were detected at the whole heart level between GT_ISO_ and WT_ISO_. Nevertheless, at cellular level, GT_ISO_ myocytes showed a significant increase of the cell size mostly due to cell widening (Figure 1 and Figure S1), confirming the early adaptative compensatory stage characterized by concentric hypertrophy and not the more advanced heart failure stage of structural remodeling. Despite the increased cell size and fibrosis in GT_ISO_, the total weight of the heart remained similar to WT_ISO_, suggesting a decreased number of myocytes in GT_ISO_ due to cell apoptosis or necrosis. While the hypertrophy program may be explained by enhanced AC/cAMP/PKA signaling caused by A4 deficiency, additionally, A4 may play a role in cell death.

The role of annexins in apoptosis has been shown to be isoform‐ and cell‐specific. While A5 and A11 are proapoptotic annexins [13, 52], antiapoptotic roles have been identified for annexins A4, A1, and A9 in cardiomyocytes and cancer cells [22, 53, 54]. A4, together with A5 and A6, is responsible for the stability and repair of the plasma membrane [6, 7], increasing the likelihood that cells deficient in A4 are more prone to membrane instability and thus cell necrosis.

Another factor that can contribute to fibrosis and structural remodeling is inflammation. It has been suggested that annexin A1 may have anti‐inflammatory effects [55, 56], while A4 interacts with p50 in a Ca^2+^‐dependent fashion to modulate NFκB signaling pathway [57] Recent studies showed that the overexpression of A4 in a myocardial ischemia reperfusion model reduced cardiomyocytes apoptosis, inflammation, and oxidative stress as well as the size of the myocardial infarction area [22] possibly due to a combined effect on NO production in macrophages and a better wound healing due to cell migration [58, 59] In line with these studies, our results show that the lack of A4 was accompanied by a decrease of *Sod2* mRNA levels due to ISO treatment, and further decrease of *Sod2* in the GT_ISO_ group, suggesting a reduced antioxidant capacity, increased ROS, and potentially more cellular damage. This effect has not been shown at the cardiac level for other members of the annexin family. The moderate upregulation of A4 detected in WT_ISO_ may have been beneficial in this respect. Thus, our results support a protective indirect mechanism mediated by annexin A4. Our study opens new questions regarding putative roles of annexin A4 in apoptosis, necrosis, inflammation, or even the hypertrophy program of cardiomyocytes.

Regarding the activation of the hypertrophy gene program, our results confirmed that ISO treatment changed the mRNA levels of three genes considered hallmark for hypertrophy such as *Nppa*, *Myh6*, and *Tnni3*, encoding for atrial natriuretic peptide, alpha myosin heavy chain, and troponin I, respectively, without changes for *Nppb* and *Myh7* encoding for brain natriuretic peptide and beta myosin heavy chain, respectively, regardless of genotype. The gene expression profile of α‐ and β‐myosin heavy chain shows that the isoform switch was not completed and indicates that our study assessed an early time point in remodeling. At the same time point, A4 gene was upregulated in WT_ISO_, as reported for patients with heart failure [23]. These data suggest that A4 deficiency does not impact ISO‐induced transcription; however, A4 may be one of the genes up‐regulated under chronic βAR stimulation. Recent studies identified increased A4 protein level in response to muscle tissue damage, both in skeletal and cardiac tissue in relation to an early compensatory response aiming to initiate regenerative mechanisms [22, 60]. Future experiments are necessary to investigate in detail the expression of annexin A4 at protein levels in relation to other annexins and other Ca^2+^‐buffering proteins in different models of cardiomyopathies. Our data show that the presence of annexin A4 is sufficient to delay ISO‐induced hypertrophy at the cellular level in WT mice without altering the transcriptional program of cardiac hypertrophy.

### 4.2 Electrical Remodeling

Little is known about the role of A4 in the cardiac electrical remodeling in pathology. Links between annexins and systolic dysfunction [26], excitation‐contraction coupling [10] or electro‐mechanical coupling [61] have been established for A5, A6, and A7, respectively. More specifically, studies have revealed interactions of different annexins with membrane proteins like L‐type calcium channel [25], Na^+^‐Ca^2+^ exchanger [10, 11] or RyR [10, 12] suggesting that annexins may modulate directly or indirectly the activities of ion channels in normal or diseased states impacting electrical remodeling.

The prolongation of the action potential (AP), the decrease of *I*_to_ and *I*_CaL_, and the increase of *I*_NCX_ are hallmarks of cardiac electrical remodeling in hypertrophy and heart failure. In the current study, these changes have been measured both in WT_ISO_ and GT_ISO_, albeit to different extents. GT_ISO_ myocytes displayed a larger increase of APD_50_ and APD_90_, mediated by a larger decrease of *I*_to_ and a smaller decrease of *I*_CaL,_ versus WT_ISO_ myocytes. Additionally, a significant increase of *I*_NCX_ was depicted only in GT_ISO_ versus control GT_NaCl_ myocytes, but not in the WT_ISO_ versus WT_NaCl_ myocytes. Similar electrophysiological changes had been reported in patients with heart failure [62], in mice genetically modified for effectors of β1‐AR signaling pathway, such as AC5 overexpression [30] or transcription factor CREM (cAMP response element modulator) overexpression [63]. The longer APD_90_ duration at baseline was reflected in the QT interval prolongation in WT_ISO_ versus WT_NaCl_ (Figure S4a). No differences were detected between GT_ISO_ and WT_ISO_. Moreover, GT_ISO_ was not different from GT_NaCl_, probably due to the higher sensitivity of GT mice to the stress induced by the implantation of the osmotic minipumps. Publications showed that in mice, due to the short AP duration and a difference in electrical conduction between the right and left ventricles [64, 65, 66], the spatiotemporal summation of AP of individual cells in the ECG is strongly influenced by several parameters and not only by the APD of single cells. Together with the early time of remodeling investigated, this explains why no significant changes were detected in the global cardiac electrical activity in ISO‐treated mice.

Action potential prolongation is a substrate for the onset of early afterdepolarization and thus a hallmark for the proarrhythmic phenotype of hypertrophic myocytes, both ventricular and atrial [42, 62]. The major currents underlying the repolarization are K^+^‐ and Ca^2+^‐currents [67], and additionally in heart failure, an important role play NCX1 current [46] and late Na^+^ current [68].

#### 4.2.1 Alterations at the Level of K^+^‐Channels/Current

Human and murine action potential differ both in shape and underlying currents [69]. Many K^+^‐channels expressed in mouse ventricular myocytes contribute to the fast AP repolarization to support the high heart rate in mice as opposed to large mammals. The molecular analysis of the expression of some K^+^‐channels' subunits (Figure 3) showed that under ISO‐treatment mRNA levels of *Kcnd3*, *Kcna4*, *Kcna5*, *Kcnb1*, and *Kcnip2* but not *Kcnd2*, decreased significantly to levels not different between WT_ISO_ and GT_ISO._ Despite the mRNA reduction, the protein levels for Kv4.3, Kv4.2, and KChIP2 were not changed, indicating that 1 week after ISO infusion, the slow turnover of the membrane proteins did not follow the transcriptional changes. On this comparable molecular background, the significant decrease of the *I*_to_ currents in GT_ISO_ versus WT_ISO_ suggests that posttranslational rather than transcriptional regulation of K^+^‐channels may explain the differences between K^+^‐currents of GT_ISO_ and WT_ISO_ myocytes. In mouse ventricular myocytes, *I*_to,f_ flows through heteromeric Kv4.2/Kv4.3 [70] as opposed to human ventricular myocytes where *I*_to,f_ flows through homomeric Kv4.3 [62, 71, 72]. Our results showing ISO‐dependent *Kcnd3* downregulation are in line with previous studies showing reduced *Kcnd3* levels and *I*_to_ in ventricular myocytes from patients with heart failure [62], and thus, relevant to human *I*_to_ and the underlying channel. It was interesting that no change of APD_20_ occurred despite a 50% reduction of the total *I*_to_ in GT_ISO_. Several findings can explain these results: (1) due to the dynamic change of the membrane potential, only a fraction of *I*_to_ current may be enough for the early repolarization; (2) the overall decrease of total *I*_CaL_ diminishes *I*_CaL_ contribution to depolarization at voltages above +20 mV; (3) other fast activating K^+^‐currents may assist repolarization. Further computer modeling of the electrical activity of ventricular myocyte may confirm the dynamic involvement of the K^+^ and Ca^2+^ currents to AP duration in these conditions.

Multiple mechanisms may be involved in *I*_to_ reduction under βAR stimulation. One hypothesis is that due to A4 deficiency in GT mice the increase of cAMP production [21] will activate PKA, an enzyme shown to decrease I_to_ current [73], most likely via indirect mechanisms affecting the surface expression of Kv4.2 [74]. Based on primary protein sequence analysis, Kv4.2 and Kv4.3 contain multiple PKA phosphorylation sites, some confirmed for Kv4.2 [75, 76], however, the association with KChIP protein is necessary for *I*_to_ inhibition due to PKA activation [77]. Additionally, the dissociation of Kv4.2 from KChIP2 following Kv4.2 phosphorylation by sympathetically activated PKA may decrease *I*_to_ [73]. Studies performed in neonatal ventricular myocytes stimulated for 48 h with ISO showed *I*_tof_ reduction and decreased mRNA expression of *Kcnd2/3* and *Kcnip2*, a reduction that could be partially prevented only by NFκB inhibition [78]. Moreover, β‐adrenergic activation increases CaMKII activity [78], as well as mitochondrial reactive oxygen species and reactive nitrogen signaling, which may affect ion channels activity [79, 80].

#### 4.2.2 Alterations at the Level of Ca^2+^‐ Transporters

The protein levels of the α‐subunit of L‐type calcium channel were unchanged due to ISO treatment, independent of genotypes, despite the decrease of mRNA levels of *Cacna1c* in ISO‐treated groups. Our data show that chronic ISO decreased *I*_CaL_ levels in both genotypes; however, in GT_ISO_ *I*_CaL_ was better preserved and the voltage dependence of activation was shifted to more negative potentials (Figure 6), in line with changes reported in failing human ventricular myocytes [81]. Both alterations are consequences of PKA‐dependent phosphorylation of the L‐type Ca^2+^‐channels. These changes induced an increase of the window *I*_CaL_ current that may explain a larger contribution of *I*_CaL_ to APD_90_ in GT_ISO_, and an increase of RT_50%peak_ of CaT in myocytes isolated from ISO‐treated mice, without differences between genotypes (Figure S5c). PKA activation may result from larger cAMP production upon release of AC5 from A4 inhibition [39]. Interestingly, cAMP levels were found decreased in ISO‐treated mice (Figure 9g), in line with reduced mRNA levels of both AC5 and AC6 (Figure 10c). These findings would suggest decreased PKA activity, which was not reflected at the phosphorylation levels of PLB and CREB (Figure S5g,h). One limitation of the study is given by the fact that our measurements reflect the global cAMP levels and they do not give information about local cAMP concentration. Recent studies demonstrated that cAMP is not uniformly distributed in cardiomyocytes, but rather concentrated in specific subcellular microdomains and thus controlling locally specific signaling pathways [82]. Since A4 is preferentially localized to the plasma membrane, it is likely that it will impact more the subsarcolemmal cAMP pool, and less the cytosolic pool that would be responsible for PLB and CREB phosphorylation. Reduced cAMP levels at similar PKA‐dependent phosphorylation levels may indicate either increased PKA sensitivity to cAMP or lower phosphodiesterase activity under prolonged stress. However, PKA may not be the only enzyme modulating L‐type channel. An alternative pathway may involve calmodulin kinase II (CaMKII) activation via a mechanism involving downregulation of Kv4.3 [83, 84]. Overall, a larger *I*_CaL_ at negative potentials measured in GT_ISO_ versus WT_ISO_ may explain the compensatory APD_50_ and APD_90_ prolongation, to provide enough Ca^2+^ for a normal contraction. This increased influx of Ca^2+^ would be required to compensate for a potentially lower SR calcium release caused by a reduced RyR2 phosphorylation by PKA.

The increase of Ca^2+^ influx may cause Ca^2+^ overload in the absence of the compensatory increased activity of the Ca^2+^ reuptake systems: sarcoplasmic/endoplasmic reticulum Ca^2+^ ATP‐ase (SERCA2a) to restore SR Ca^2+^ load, and the Na^+^/Ca^2+^ exchanger (NCX1) to transport Ca^2+^ to the extracellular space. The protein levels of NCX1 increased by 50%, while the mRNA levels of *Slc8a1*, respectively, were not changed significantly in ISO‐ versus NaCl‐treated groups. NCX1 upregulation was reported in heart failure as a compensatory mechanism for the downregulation of SERCA2a [67, 85]. A link between annexins and NCX1 has been reported following overexpression of annexins A5 and A6 [10, 86]. Due to the conserved calcium binding sites among annexin isoforms, annexins are regarded as “Ca^2+^‐regulated membrane binding modules” [87]. Therefore, annexin A4 may play a role as a regulator of membrane‐restricted Ca^2+^ dependent activities. In this study, the higher level of *I*_NCX_ and a faster kinetic of inactivation were changes significant for GT_ISO_ versus GT_NaCl_, thus *I*_NCX_ can be another contributor to APD prolongation, as suggested in other studies [46, 88]. Increased *I*_NCX_ could not be explained based on the increase of NCX1 protein levels or of SR Ca^2+^‐load since both were elevated to similar levels in WT_ISO_ and GT_ISO_. Overall, these results suggest rather that *I*_NCX_ increases in GT_ISO_ as a consequence of a decreased Ca^2+^ buffering capacity at subsarcolemmal level due to A4 deficiency, thus increasing the local free Ca^2+^ available to NCX1 for a faster exclusion. Higher activity of NCX1 is a substrate for arrhythmia, thus further detailed studies of Ca^2+^‐homeostasis regulation will be required to address the impact of A4‐deficiency on the excitation‐contraction coupling.

Few basic experiments that measured calcium transients and contractility exposed some differences between GT and WT. Firstly, the decreased diastolic Ca^2+^ levels in GT_NaCl_ (Figure S5a) correlate better with a faster activity of NCX1 (Figure 7c) rather than SERCA, since PLB phosphorylation was unchanged (Figure S5g). We could speculate that in ISO‐treated myocytes, the prolonged RT_50%peak_ was primarily influenced by decreased *I*_CaL_, faster calcium reuptake could be influenced either by SERCA if phospholamban is shifted to the pentamer pool either by chronic ISO‐stimulation via PKA or via CaMKII, or by NCX1, while the decreased contractility indicates the onset of failure. Moreover, the increase of baseline Ca levels in GT_ISO_ versus GT_NaCl_, could be a consequence of CaMKII activation and RyR phosphorylation which would increase SR Ca leak. All these mechanisms need to be confirmed by future experiments. Overall, chronic ISO treatment did not induce genotype‐dependent effects on calcium handling or contractility.

#### 4.2.3 Response to Acute ISO Stimulation

Sustained hyperactivity of the sympathetic system leads to a reduced response of failing hearts to βAR agonists, likely caused by a desensitization of βAR due to uncoupling from G‐proteins, β‐arrestin‐dependent receptor internalization [89, 90] or the downregulation of adenylyl cyclases [91]. Figure 8b shows that after 7 days of chronic ISO infusion, WT_ISO_ and GT_ISO_ myocytes responded to ISO by APD_20_ prolongation, while the prolongation of APD_90_ was blunted in ISO‐treated groups independent of genotype. The response may depend on the signaling complexes organization in lipid rafts including AC5 or AC6, PKA and either K^+^‐channels or Ca^2+^‐channels. Taking into consideration that *I*_to_ are K^+^‐currents involved in the first phases of repolarization [92] reflected in APD_20_ and that Ca^2+^‐ currents contribute more to APD_90_ prolongation under acute ISO stimulation [39], our results indicate that the signaling mechanisms activated via βAR stimulation are capable of regulating K^+^‐channels but fail to interact with L‐type calcium channels. Multiple mechanisms have been proposed to explain the loss of L‐type channel regulation by PKA, such as α1C proteolysis of C‐terminus, altered expression of A‐kinase anchor proteins to the signaling complex, or disruption of interaction between β2 auxiliary subunit and α1C [93, 94] Together with the positive chronotropy detected in the heart rate (Figure 8c), the positive inotropy (Figure 9f) and lusitropy (Figure 9e), these results show that βAR responsiveness was reduced, but still present at the investigated time point. At the time point of our investigation, despite the alterations in APD due to acute ISO stimulation, we did not detect changes in the global electrical activity of the heart and in the incidence of ventricular extrasystoles.

In response to acute ISO stimulation, cAMP production increases in NaCl‐treated mice. A4‐deficient cardiomyocytes chronically treated with ISO displayed a significant increase in cAMP production in response to acute ISO stimulation. In WT_ISO_ versus GT_ISO_, where A4 may be expressed at least at physiological levels, RT_50%peak_ and cAMP production were not changed by acute ISO stimulation (Figure 9d,g). The increased expression of A4 (Figure 1e) and decreased expression of AC5 (Figure 10c) unmask an increased A4/AC5 ratio in WT_ISO_, thus more AC5 inhibition will produce less cAMP under acute stress (Figure 9f). Consequently, as this is a cAMP subsarcolemmal pool it is likely to decrease the subsarcolemmal PKA activity that would phosphorylate L‐type calcium channel, preventing *I*_CaL_ increase and thus the decrease of the rise time of the CaT (Figure 9d). A4 deficiency modifies subsarcolemmal cAMP signaling during chronic β‐adrenergic stimulation.

Overall, chronic ISO treatment reduced the response to acute ISO, whereas no additional genotype‐dependent effect on electrical activity, calcium handling, or contractility was observed. Thus, our study opens new research directions regarding the long‐term involvement of annexins in cardiac pathology.

## 5 Pathological Relevance

Annexin A4 emerges as a potential therapeutic tool to specifically block AC5, while preserving the cardiac protective effect of a functional AC6. The therapeutic use of peptides derived from annexins has been shown for annexin A1, where the active peptide chain Ac2‐26 can promote wound healing or reduce the susceptibility to AF in obese mice by decreasing atrial fibrosis, lipid deposition, oxidative stress injury, and myocardial cell apoptosis [22, 56, 95]. Successful clinical trials reported the use of TAT‐conjugated neuroprotective peptide (NA1) [96] or an adenovirus 5 encoding AC6 in heart failure patients [97]. Moreover, mice intravenously injected with AAV9 carrying A4 gene displayed reduced myocardial damage due to ischemia/reperfusion lesion [22]. Previously we have shown that the N‐terminal A4 peptide TAT‐A4N_1–22_ was sufficient to limit ISO‐induced APD prolongation in GT myocytes in vitro [39]. Future in vivo experiments will be necessary to test the potential of A4 N‐terminal peptide to impair the progression of the pathological remodeling induced by sympathetic hyperactivity in the heart.

## 6 Conclusions

Our findings show that the lack of annexin A4 facilitates the progression of electrical and structural remodeling toward a pro‐arrhythmic phenotype. Most of the changes depicted in GT_ISO_ can be explained based on an enhanced activation of the AC/cAMP/PKA pathway. The upregulation of A4 in failing human hearts [23] or ischemia/reperfusion [22] may be part of a protective compensatory mechanism, aiming to negatively modulate βAR signaling at the level of AC. One limitation of our study is that it does not show A4's protection against long‐term heart failure progression or mortality. It is likely that A4 upregulation is limited with heart failure progression; thus, along with other recently published studies, we propose that a therapeutical elevation of A4 levels via A4 delivery may be beneficial to reduce the ISO‐induced cardiac damage.

## Author Contributions

Frank U. Müller, Alexander Heinick, Florentina Pluteanu, and Christina Hermes conceived and designed the research; Florentina Pluteanu, Manuel Domnik‐Lehnert, and Christina Hermes performed the research and acquired the data. Florentina Pluteanu, Alexander Heinick, Manuel Domnik‐Lehnert, and Christina Hermes analyzed and interpreted the data. All authors were involved in drafting and revising the manuscript.

## Funding

The study was supported by IZKF Munster (Mu1/011/17 to FUM) and the DFG (Mu1376/11‐3 to FUM).

## Disclosure

During the preparation of this work, the author(s) did not use Generative AI and AI‐assisted technologies in the writing process.

## Conflicts of Interest

The authors declare no conflicts of interest.

## Supporting information


**Figure S1:** The effect of chronic ISO stimulation on cell width. Bar graphs show mean ± SEM, *N* = 4 mice. *p < 0.05 vs. control (gray vs. GT_NaCl_), ^#^ *p* < 0.05 GT_ISO_ vs. WT_ISO_ (two‐way ANOVA, Holm‐Sidak post‐test).
**Figure S2:** Alterations of L‐type window current due to chronic ISO‐infusion. (a) Window current of *I*_CaL_ resulted from the product of steady state inactivation and activation curves in the four experimental groups. The dots show the mean values. The window current awas integrated to calculate the area under the curve (AUC) represented in panel (b), the voltage where maximum is reached, *V*_max_ (c) and the amplitude at maximum, *Y*_max_ (d). Bar graphs show mean ± SEM from *n*/*N*: 18/7 (WT_NaCl_), 18/7 (GT_NaCl_), 17/9 (WT_ISO_), 13/7 (GT_ISO_) where *n* is the number of cells and *N* is the number of mice. *p < 0.05 (as shown), where gray was used to compare GT groups, red ISO‐treated groups; (one‐way ANOVA, Holm‐Sidak post‐test b–d).
**Figure S3:** Western blots of Kv4.2, Kv4.3, KChIP2, LTCC, and NCX and the corresponding Ponceau stain. The red boxes show the representative bands used in Figures 5–7.
**Figure S4:** Duration of QT, PR and QRS interval measured from the ECG measurements for the four experimental groups. QT interval measured at baseline (a), while PR (b), and QRS (c) were measured before and after acute ISO injection. Data show mean ± SEM. *N* = 8 mice per group. *p < 0.05 and **p < 0.01 as shown, one way ANOVA, Holm Sidak post hoc test.
**Figure S5:** Excitation‐contraction coupling and phosphorylation level of PKA targets at baseline.

## Data Availability

The data that support the findings are available in the article and its Supporting Informaton. Further details can be requested via email from the corresponding and first authors.
