# Supplementary material for: Annexin A4 Deficient Ventricular Myocytes Display Advanced Hypertrophy and Electrical Remodeling With Chronic β‐Adrenoceptor Stimulation
Source: FASEB J. 2026 Sep 2;40(17):e72213. doi: 10.1096/fj.202600257R (PMC13537368; doi:10.1096/fj.202600257R)
Supplement: Supplementary file 1 [file FSB2-40-e72213-s001.docx]

**Annexin A4 deficient ventricular myocytes display advanced hypertrophy and electrical remodeling with chronic β adrenoceptor stimulation**

Florentina Pluteanu^a,b,1,*^, Alexander Heinick^b,1^, Manuel Domnik-Lehnert^b^, and Frank U. Müller^b^, Christina Hermes^b,*^

^a^Department of Anatomy, Animal Physiology and Biophysics, Faculty of Biology, University of Bucharest, 91-95 Spl. Independenţei, 050095 Bucharest, Romania;

^b^Institute of Pharmacology and Toxicology, Universität Münster, Domagkstr. 12, D-48149 Münster, Germany

**Supplemental Data**

All experiments involving commercially available products were performed according to the manufacturer’s specifications. Most methodological procedures had been described previously.[1, 2]

*Experimental animals*

FVB/N mice deficient in annexin A4 (anxa4a^-/-^) and the corresponding phenotype were previously described.[3] Male anxa4a^-/-^ (GT, gene trapped) and wild type anxa4a^+/+^(WT) littermates were used at 19-23 weeks of age. Mice were kept at a room temperature of 22°C, under a 12-h light/dark cycle, received normal diet (Altromin Spezialfutter GmbH, Lage, Germany) and water ad libitum. All procedures were conducted in accordance with local animal welfare authorities and approved by the Landesamt für Natur, Umwelt und Verbraucherschutz Nordrhein Westfalen, Germany (Ref. no. 84_02.04.2015.A504).

FVB/N mice deficient in annexin A4 (anxa4a-/-, GT) and the corresponding phenotype were previously described. Male anxa4a-/- (GT, gene trapped) and wild type anxa4a+/+(WT) littermates were used at 19-23 weeks of age. A total number of 37 GT and 36 WT mice have been used for this study. Mice were kept at a room temperature of 22°C, under a 12-h light/dark cycle, received normal diet (Altromin Spezialfutter GmbH, Lage, Germany) and water ad libitum. The mice were euthanized with CO2 inhalation. All procedures were conducted in accordance with local animal welfare authorities and approved by the Landesamt für Natur, Umwelt und Verbraucherschutz Nordrhein Westfalen, Germany (Ref. no. 84_02.04.2015.A504).

*Chronic isoproterenol treatment*

Osmotic mini‐pumps (model 2001, Alzet, Cupertino, CA, USA) were implanted into GT and WT mice for continuous application of 30 mg/kg body weight/day (30 mg kg−1xd−1) of isoproterenol (ISO) (DL‐isoproterenol hydrochloride, Merck, Darmstadt, Germany) dissolved in a solution containing 2 mM HCl in 0.9% of NaCl. The osmotic mini-pumps treatment lasted for 7 days and was used according to the manufacturer's instructions. ISO – treated mice were grouped according to genotype into ISO-treated GT (GT_ISO_) and ISO-treated WT (WT_ISO_). In control animals, the osmotic mini-pumps contained only the solution with 2 mM HCl in 0.9% of NaCl and created the NaCl-treated groups of GT or WT (GT_NaCl_ and WT_NaCl_, respectively). Animals of different genotypes were randomly assigned to receive either NaCl or ISO treatment.

*Heart size*

Mice were euthanized with CO_2_ and hearts were excised. Hearts were cleaned from tissue remnants, washed with 0.9% NaCl solution and patted dry. The hearts were weighed on a precision scale and further normalized to the corresponding tibia length.

*Isolation of adult mouse cardiomyocytes*

Mouse cardiomyocytes were isolated from adult mice from all four experimental groups (WT_NaCl_, GT_NaCl_, WT_ISO_, GT_ISO_) by enzymatic digestion[1] Briefly, excised hearts were rinsed with heparin and retrogradely perfused with collagenase solution (collagenase type II; Worthington Biochemical, Lakewood, NJ, USA) in a modified Langendorff apparatus (37°C, 2 ml/minute). The cells were then separated by mechanical dispersion, and Ca^2+^ concentration was progressively increased to 1 mM before initiating the experiments.

*Cell size*

Isolated ventricular myocytes were imaged with an inverted microscope (Nikon Eclipse Ti S, Nikon Instruments Inc. Tokio Japan). Field images were captured and analyzed using NIS-Elements BR Analysis 4.13.05 software. Cell length, width and area were measured from a minimum of 50 cardiomyocytes per mouse, from N = 4 mice in each experimental group. Averages per mouse of the cell parameters were further used for statistical comparison between groups.

*Fibrosis Masson Goldner staining*

By using the Masson-Goldner staining method, connective heart tissue containing collagen is stained in blue in histological specimens. Heart slices were deparaffinized and fixed at room temperature in Bouin Solution. Picric acid staining was washed with double distilled water (ddH2O). before staining with a mix of iron hematoxylin A and iron hematoxylin B in the ratio 1 + 1. After washing with ddH2O, the slices were incubated in Ponceau acid fuchsin azophloxine solution and washed in 1% acetic acid. Then slices were differentiated using tungstophosphoric acid orange G solution and washed in 1% acetic acid. After staining the slices were dehydrated for 3 min in ascending ethanol series, followed by clarification with 2 times for 3 minutes xylene incubations. At the end the slides were sealed with a xylol-soluble fixing agent. Adjacent images from the same slice were taken with 20-fold magnification using a Nikon Eclipse Ti S microscope and were analyzed digitally by stitching them to single pictures using NIS-Elements BR Analysis 4.13.05 Software. For each generated picture was measured further the total number of pixels and the number of blue pixels using the Image-Pro Analyzer V 7.0 software

*Western blot*

Ventricles were homogenized in lysis buffer containing 100 mM NaCl, 20 mM Tris Base, 1% sodium deoxycholate, 0.1% SDS, 1% Triton X-100, pH adjusted to 8.0 and supplemented with phosphatase and protease inhibitor tablets (A32959, ThermoFisher Scientific, Germany). Forty µg of protein of each sample were equally distributed and loaded on the 10% SDS-PAGE gel. Equal protein load was assessed after transfer using Ponceau stain. The following rabbit polyclonal primary antibodies were used: Kv4.2 (1:200, APC 023 Alomone Labs, Israel), Kv4.3 (1:200, APC 017 Alomone Labs, Israel), KChiP2 (1:200, sc-25685, Santa Cruz, USA) phospho-CREB (pCREBSer133, 1:1000, Cell Signaling Technology Europe B.V., Frankfurt am Main, Germany), total CREB (CREB, 1:100, Cell Signaling Technology Europe B.V., Frankfurt am Main, Germany), phospholamban phosphorylated at Serine-16 (pPLBSer161:1000, Badrilla Ltd, Leeds, UK), calsequestrin (CSQ, 1:2500, ThermoFisher Scientific, Germany), α1C subunit of L-type calcium channel (LTCC, 1:200, Alomone LabsTM, Israel), and the monoclonal antibody for total phospholamban, clone A1 (PLBtotal, 1:1000, Badrilla Ltd, Leeds, UK), sodium calcium exchanger (NCX1, 1:1000, SWant, Switzerland). As secondary antibodies we used HRP conjugated anti-rabbit IgG antibody (1:10000, ThermoFisher Scientific, Germany) anti-mouse IgG antibody (1:10000, Sigma Aldrich, Steinheim, Germany), further developed with SERVALight Helios (42587.03, SERVA Electrophoresis GmbH, Germany). Band intensities were quantified using ImageJ 1.49v (NIH, Bethesda, MD, USA). Each band was normalized to Ponceau for load. before the normalization of the phosphorylated protein to the total protein. The normalized values of the samples belonging to the same group were averaged for statistical comparison using 2-way ANOVA with Holm-Sidak post-test.

*qRT-PCR*

Total RNA was isolated from cardiac ventricles of mice from the four experimental groups using the TRIzol reagent (Life Technologies, Darmstadt, Germany). Total RNA (1 μg) was reversely transcribed to cDNA using the first strand cDNA synthesis kit for qRT-PCR AMV (Roche Diagnostics, Cologne, Germany). Detection of particular cDNAs was performed with the LightCycler 1.5 System (Roche Diagnostics, Cologne, Germany) and LightCycler Fast Start DNA Master SYBR Green I Mix (Roche Diagnostics, Cologne, Germany), as described before,[4] using specific mouse primers for:

**annexin A4** (*Anxa4*):

5’ AGCGCCAGGAGATCAGGA 3’ (fwd),

5’ ATATTGCTGCTGGTATGT 3’ (rev);

**natriuretic peptide A** (*Nppa*):

5’-GGGGGTAGGATTGACAGGAT-3’ (fwd),

5’-CAGAGTGGGAGAGGCAAGAC-3’ (rev);

**natriuretic peptide B** (*Nppb*):

5’-GACCAAGGCCTCACAAAAGA-3’ (fwd),

5’-AGACCCAGGCAGAGTCAGAA-3’ (rev);

**myosin heavy chain 6** (*Myh6*):

5’-GATGCCCAGATGGCTGACTT-3’ (fwd),

5’- GGTCAGCATGGCCATGTCCT-3’ (rev);

**myosin heavy chain 7** (*Myh7*):

5’- GCCAACACCAACCTGTCCAAGTTC-3’ (fwd),

5’- TGCAAAGGCTCCAGGTCTGAGGGC -3’ (rev);

**troponin I3, cardiac type** (*Tnni3*):

5’-GACTTATGCCGACAGCTTCA-3’ (fwd),

5’-TTGCCACGGAGGTCATAGAT-3’ (rev);

**α1C subunit of L-type calcium channel** (*Cacna1c*):

5’-GCTCTCTTCACCGTCTCCAC-3’ (fwd),

5’-GACGAAACCCACGAAGATGT-3’ (rev);

**potassium voltage-gated channel subfamily D member 2** (*Kcnd2*):

5’-CTGCTCACGGAGACACAAAA-3’ (fwd),

5’-CGGCTGTTGGATAGTGGAGT-3’ (rev);

**potassium voltage-gated channel subfamily D member 3** (*Kcnd3*):

5’-TGTACGAACCTCCACCATCA-3’ (fwd),

5’-AGTGGCTGGACAGAGAAGGA-3’ (rev);

**potassium voltage-gated channel interacting protein 2** (*Kcnip2*):

5’-GACATGATGGGCAAGTACACC-3’ (fwd),

5’-ACGCCGTCCTTGTTTCTGT-3’ (rev);

**potassium voltage-gated channel subfamily A member 4** (*Kcna4*):

5’-GAAGAAGGGGTCAAGGAATC-3’ (fwd),

5’-TGGCAGGTGGAGAGAACAAT-3’ (rev);

**potassium voltage-gated channel subfamily A member 5** (*Kcna5*):

5’-TCCGACGGCTGGACTCAATAA-3’ (fwd),

5’-CAGATGGCCTTCTAGGCTGTG-3’ (rev);

**potassium voltage-gated channel subfamily B member 1 (*Kcnb1*):**

5’-CCACCAGATTCTCCCACAGT-3’ (fwd),

5’-GCTCTCCACGAAGAAACCAG-3’ (rev);

**adenoreceptor beta-1 (***Adrb1***):**

5’ GCTGCAACGCTCACCA 3’ (fwd),

5’ GCAGGAGCAATGATCTTGATCT 3’ (rev);

**adenoreceptor beta-2 (***Adrb2***):**

5’ CACAGCCATTGCCAAGTTGG 3’ (fwd),

5’ CGGGCCTTATTCTTGGTCAGC 3’ (rev);

**adenylyl cyclase 5 (***Adcy5***):**

5’ ACGATTGGTAGTACAGCTCGTCATTGCGCC 3’ (fwd),

5’ TGCTCAGCCTGCTGGCCTGCTCCCGTGTTCC 3’ (rev**);**

**adenylyl cyclase 6 (***Adcy6***):**

5’ GCCATGTAGGTGCTACCG 3’ (fwd),

5’ GAGGCAAACAACGAGGGC 3’ (rev);

**G-alpha subunit I (***Gnai1***)**

5’ GCAAGATGATCGACCGCAAC 3’ (fwd),

5’ GACCACTGCCTTGTACTGCT 3’ (rev);

**G-alpha subunit II (***Gnai2***):**

5’ CACGGCCATCATCTTCTGTG 3’ (fwd),

5’ AGGGAAACAGATGGTCAGGG 3’ (rev);

**G-alpha subunit III (***Gnai3***)**

5’ GGACGGCTATTCAGAGGACG 3’ (fwd),

5’ TAAAGCATGCCTGTACCCCG 3’ (rev);

**G-alpha subunit s (***Gnas***)**

5’ ACAGTAAGACCGAGGACCAG 3’ (fwd),

5’ CCTTCTCACCATCGCTGTTG 3’ (rev);

**superoxide dismutase 2 (***Sod2***):**

5’-ACAACTCAGGTCGCTCTTCA-3’ (fwd),

5’-TCCTTGCAATGGGTCCTGAT-3’ (rev);

**glyceraldehyde 3 phosphate dehydrogenase (***Gapdh***) as housekeeping gene:**

5’- AACTTTGGCATTGTGGAAGG-3’ (fwd),

5’-GGATGCAGGGATGATGTTC-3’ (rev).

Relative gene expression was calculated using the 2-DDCT method incorporated in the relative expression software tool REST, ver. 2.013[5] using *Gapdh* as housekeeping gene and relative to WT_NaCl_.

*Cellular electrophysiology*

Ca^2+^ tolerant ventricular myocytes (VM) were used for the measurements of action potentials[6] and current[7] using amphotericin B perforated whole cell patch clamp method. All recordings were performed at room temperature (22-24°C, RT). Borosilicate glass capillaries (Science Products, Hofheim, Germany) were pulled to a resistance of 3 5 MΩ. Data were acquired and filtered at 10 kHz using an EPC 800 amplifier and sampled with an 18bit A/D converter InstruTech ITC 18 under the control of the PatchMaster software (HEKA Elektronik, Lambrecht, Germany). At least 55 % compensation of series resistance was set in voltage clamp experiments. For the measurement of action potentials and K^+^-currents, the bath solution contained (in mM): 136 NaCl, 5.4 KCl, 1.8 CaCl_2_, 1 MgCl_2_, 5 HEPES, 0.33 NaH_2_PO_4_ and 10 glucose, pH 7.4, and the pipettes were filled with a solution containing (in mM): 5 NaCl, 120 KCl, 2.5 MgATP, 1 EGTA and 5 HEPES pH 7.4. For L-type Ca^2+^ current measurements bath solution was supplemented with 100 mM BaCl_2_ and 10 mM TEA-Cl to block background and slow K^+^ currents, respectively, and the pipettes were filled with a Cs^+^ based solution containing (in mM): 120 CsCl, 1 MgCl_2_, 5 Na_2_ATP, 10 TEA-Cl, 10 EGTA and 10 HEPES, pH 7.2. The NCX current (INCX) and the calcium stores of the sarcoplasmic reticulum (SR Ca^2+^ load) was measured using a 10 mM caffeine pulse, using a pipette solution containing (in mM): 120 CsCl, 20 TEA-Cl, 5 Na_2_ATP, 10 HEPES, 5 MgCl_2_ and 0.2 EGTA, pH 7.2, and the bath solution containing (in mM): 135 NaCl, 5.4 KCl, 1 MgCl_2_, 1.8 CaCl_2_, 10 HEPES, 10 glucose, 5 4-aminopyridine 0.1 BaCl_2_, pH 7.4.[8].

Action potentials (AP) were recorded in the current clamp mode in ventricular myocytes stimulated for 20 s at 1 Hz frequency using supra-threshold stimuli of 3-6 ms duration. Three to five consecutive AP traces at steady state were averaged and AP duration was measured from the peak to 20, 50 and 90 % repolarization.[6] The signal corresponding to the stimulus has been offline subtracted for the clarity of representation. To measure the total outward K^+^-currents, the cells were clamped at -80 mV followed by a 10 ms prepulse to -40 mV to inactivate the sodium channel and a series of 500 ms voltage steps from -80 to +70 mV in 10 mV increment. The transient outward channel (I_to_) was inactivated in a second voltage protocol consisting of a 180 ms prepulse to -40 mV followed by a 5 s voltage steps from -40 to +70 mV in 10 mV increment. The current amplitudes measured at the beginning and at the end of the depolarizing pulse in the two voltage protocols correspond to I_Kpeak_ and I_Kslow_, and I_Kend_ and I_Kss_, respectively. Background current at -40 mV was subtracted from the current amplitudes before normalization to the membrane capacitance. The Ito currents were obtained after offline subtraction of the current traces corresponding to the depolarizing epochs of the two voltage protocols. Ito amplitudes were measured as peak current minus the current at the end of the subtracted trace and were normalized to the membrane capacitance. The inward rectifier I_K1_ current was measured as the current at the end of the 500 ms pulses between -120 and -10 mV elicited from -40 mV holding voltage. To record voltage gated Ca^2+^ currents (I_CaL_), voltage protocols consisted of 400 ms activation steps between -55 to + 45 mV in 5 mV increment were applied from a prepulse potential of –40 mV (to inactivate Na^+^ currents) and were followed by a 300 ms step to 0 mV to evaluate the remaining fraction of closed channels (inactivation step). Between pulses, the holding voltage was maintained at – 80 mV. I_CaL_ amplitude, calculated as peak amplitude minus the current amplitude at the end of each pulse, was normalized to membrane capacitance to obtain the current density (pA/pF). The cells that showed more than 20% I_CaL_ desensitization during the first 10 minutes of recording were not used for further analysis. Current-voltage (IV) relations, voltage dependence of activation and inactivation were calculated as described before.[7] To record I_NCX_ and SR Ca^2+^-load, myocytes were stimulated at 1 Hz via the pipette for at least 1 min using 200 ms depolarizing pulses from -80 mV to 0 mV prior to the application of 10 mM caffeine. Baseline current was subtracted, and the current trace was analyzed for peak amplitude and the kinetic of decay to estimate NCX activity. The integrated area delineated by the caffeine-induced inward current was an estimation of SR Ca^2+^-load. Acute stimulation with 1mM ISO was achieved by topical application using a gravity driven perfusion system with a 0.5 mL/minute flow rate. A stock solution of 1 mM ISO in 5 mM ascorbic acid was daily prepared, kept in dark on ice, and it was freshly diluted to 1 µM in the bath solution prior to application. The cardiomyocytes were kept in normal bath solution with 1 mM CaCl_2_ at room temperature and were used for recordings for up to 5 hours after isolation. For recordings we used quiet ventricular myocytes presenting clear striations, rectangular shape. All salts and reagents were purchased from Sigma Aldrich (Steinheim, Germany). The investigator was blinded to the group allocation both during recording and analysis of the study.

*Ca transients and sarcomere length shortening*

Calcium transients (CaT) and sarcomere length (SL) were measured simultaneously in single cardiomyocytes using a Nikon Eclipse Ti-S microscope, a MyoCam-S camera (IonOptix, USA), and IonWizard 6.4.1.73 software. Cells were loaded with the ratiometric dye Indo-1 AM (Invitrogen) for 10 min in the dark in the presence of Pluronic to enhance dye uptake, followed by 5 min for de-esterification. Indo-1exhibits fluorescence shift depending on calcium binding (~400 nm bound, ~475 nm unbound) upon excitation at ~340 nm, reflecting intracellular calcium levels.

Cells were placed in a Tyrode-filled perfusion chamber, settled for 5 min, and washed to remove excess dye under continuous perfusion. Electrical field stimulation (2 ms duration, 20 V, 0.5 Hz) was applied for 10 min using a MyoPacer Cell Stimulator (IonOptix) before measurement. Baseline calcium transients and sarcomere shortening were recorded from 10–12 cells under basal conditions and after acute isoproterenol (1 μM) stimulation. The ratio of fluorescence emission (405 nm/495 nm) indicates the variation of the cytosolic Ca^2+^ concentration and was analyzed for baseline, amplitude and kinetic of Ca^2+^ uptake. SL changes were analyzed using fast Fourier transform-based software to estimate the fractional shortening (%FS) as percentage of diastolic SL length. Data analysis was performed with IonWizard 6.4.1.73.

*cAMP measurements*

Cardiomyocytes from each experimental group were treated with isoproterenol (1μM) or vehicle (water) in PBS for 5 minutes at 37°C and in 5 % CO_2_, as previously described [1]. The cells were lysed and the supernatant containing the cytosolic fraction was used for cAMP measurements using the Amersham cAMP Biotrak enzyme immunoassay (ELISA, GE Healthcare, Little Chalfont, United Kingdom), starting from an input volume corresponding to 0.3 µg protein. The optical density was detected at 450 nm within 30 minutes using a Mithras LB 940 Microplate Analyzer (Berthold Technologies, Bad Wildbad, Germany). After subtracting the optical density (OD) corresponding to the non-specific binding, the sample OD was normalized to the OD of the zero standard, and extrapolated from the cAMP standard curve to derive the cAMP concentration. For each experimental design, the cAMP levels were expressed as cAMP quantity normalized to the total protein content of the input volume.

*ECG measurements*

Electrocardiograms were acquired from 7-8 mice for each of the four experimental groups, as previously described (Heinick et al, 2020). Briefly, the mice were anesthetized by inhalation with isoflurane-nitrous mixture, according to the approved animal protocol. Mice were positioned supine on a 37°C heating plate and 5 subcutaneous limb electrodes were placed after loss of toe pinch reflex. For electrocardiogram recordings electrodes were connected over an external biological amplifier (Dual Bio Amp, ADInstruments, Dunedin, New Zealand) to a data acquisition unit (PowerLab 2/20, ADInstruments, Dunedin, New Zealand), which recorded lead I and II and calculated lead III using LabChart 7 Pro software (ADInstruments, Dunedin, New Zealand). The protocol included a 5 min recording-phase under basal condition followed by an additional 30 min recording-phase after intraperitoneal injection of isoproterenol (2 mg/kg body weight). Lead II was used for the estimation of heart rate (HR), PR interval and QT interval measured before and after acute ISO application.

*Statistical analysis*

Data are presented as mean ± SEM; where n is the number of independent samples or cells, and N is the number of animals. The normal distribution was tested using Shapiro-Wilk normality test. For two group comparisons we used two-tailed unpaired parametric or nonparametric t test according to the Gaussian distribution. For multi group comparison two-way ANOVA or two-way repeated measures (RM) ANOVA were followed by Holm-Sidak post hoc test. The sample size for the single cell measurements was selected to yield 80% statistical power at the significance level of α=0.05. GraphPad Prism version 5.0 (GraphPad Software, La Jolla California, USA) and SigmaPlot 11.2 (Systat Software, San Jose, CA) were used for statistical analysis. Data were considered significantly different at P<0.05.

**Supplementary figures**

**Figure S1**

**Fig. S1: The effect of chronic ISO stimulation on cell width.** Bar graphs show mean ± SEM, N=4 mice . *P<0.05 vs. control (grey vs. GT_NaCl_), ^#^P<0.05 GT_ISO_ vs. WT_ISO_ (two-way ANOVA, Holm-Sidak post-test)

**Figure S2**

**Fig. S2: Alterations of L-type window current due to chronic ISO-infusion.** (a) Window current of I_CaL_ resulted from the product of steady state inactivation and activation curves in the four experimental groups. The dots show the mean values. The window current awas integrated to calculate the area under the curve (AUC) represented in panel (b), the voltage where maximum is reached, V_max_ (c) and the amplitude at maximum, Y_max_ (d). Bar graphs show mean ± SEM from n/N: 18/7 (WT_NaCl_), 18/7 (GT_NaCl_), 17/9 (WT_ISO_), 13/7 (GT_ISO_) where n is the number of cells and N is the number of mice. *P<0.05 (as shown), where grey was used to compare GT groups, red ISO-treated groups; (one-way ANOVA, Holm-Sidak post-test b-d)

**Figure S3**

**Fig. S3 Western blots of Kv4.2, Kv4.3, KChIP2, LTCC and NCX and the corresponding Ponceau stain.** The red boxes show the representative bands used in Fig. 5, Fig. 6 and Fig. 7.

**Figure S4**

**Fig. S4: Duration of QT, PR and QRS interval measured from the ECG measurements for the four experimental groups.** QT interval measured at baseline (a), while PR (b), and QRS (c) were measured before and after acute ISO injection. Data show means ±SEM. N = 8 mice per group. *P<0.05 and **P<0.01 as shown, one way ANOVA, Holm Sidak post hoc test.

**Figure S5**

**Fig. S5 Excitation-contraction coupling and phosphorylation level of PKA targets at baseline**

Panels show intracellular calcium at rest expressed as Indo-1 F_400_/F_475_ ratio (a), calcium transient amplitude expressed as percentage increase above baseline (b); rise time to 50%peak (c); kinetics of calcium reuptake, expressed as time constant (τ) of decay (d), contractility expressed as percentage of SL fractional shortening of diastolic SL length (%FS, e) and basal cAMP levels (f) in cardiomyocytes isolated from the four groups. Data show mean ± SE; number of cells/N animals was 50/5 (WT_NaCl_), 50/5 (GT_NaCl_), 60/6 (WT_ISO_) and 70/7 (GT_ISO_) for panels a – e, and number of cell lsysate from N mice 7 (WT_NaCl_), 7 (GT_NaCl_), 8 (WT_ISO_) and 8 (GT_ISO_) for panel f.

Phospholamban (PLB) and cAMP response element binding protein (CREB) are phosphorylated by PKA at Ser16 and Ser133, respectively. Next panels show Western blot analysis of the phosphorylated proteins normalized to total protein for pPLB_Ser16_/tPLB (g) and pCREB_Ser133_/tCREB (h) are presented in panels g Data show mean ± SD. N mice between 6-9.

*P<0.05 and **P<0.01, ***P<0.001 as shown, one way ANOVA, Holm Sidak post hoc test.

**References**

1.	Heinick, A., et al., *Annexin A4 N-terminal peptide inhibits adenylyl cyclase 5 and limits beta-adrenoceptor-mediated prolongation of cardiac action potential.* FASEB J, 2020. **34**(8): p. 10489.

2.	Pluteanu, F., et al., *Inward Rectifier K(+) Currents Contribute to the Proarrhythmic Electrical Phenotype of Atria Overexpressing Cyclic Adenosine Monophosphate Response Element Modulator Isoform CREM-IbDeltaC-X.* J Am Heart Assoc, 2020. **9**(23): p. e016144.

3.	Li, B., J.R. Dedman, and M.A. Kaetzel, *Intron disruption of the annexin IV gene reveals novel transcripts.* J Biol Chem, 2003. **278**(44): p. 43276.

4.	Heinick, A., et al., *Annexin A4 is a novel direct regulator of adenylyl cyclase type 5.* FASEB J, 2015. **29**(9): p. 3773.

5.	Pfaffl, M.W., G.W. Horgan, and L. Dempfle, *Relative expression software tool (REST) for group-wise comparison and statistical analysis of relative expression results in real-time PCR.* Nucleic Acids Res, 2002. **30**(9): p. e36.

6.	Seidl, M.D., et al., *Characterization of the Genetic Program Linked to the Development of Atrial Fibrillation in CREM-IbDeltaC-X Mice.* Circ Arrhythm Electrophysiol, 2017. **10**(8).

7.	Pluteanu, F. and L.L. Cribbs, *T-type calcium channels are regulated by hypoxia/reoxygenation in ventricular myocytes.* Am J Physiol Heart Circ Physiol, 2009. **297**(4): p. H1304.

8.	Pluteanu, F., et al., *Early subcellular Ca2+ remodelling and increased propensity for Ca2+ alternans in left atrial myocytes from hypertensive rats.* Cardiovasc Res, 2015. **106**(1): p. 87.
